# Space-time signatures of surface warming accelerations since 1970

**DOI:** 10.1038/s41467-026-77514-z

**Published:** 2026-09-22

**Authors:** Claudie Beaulieu, Adelicia Johnson, Rebecca Killick, John Lanzante, Thomas Knutson

**Affiliations:** 1 Ocean Sciences Department, University of California Santa Cruz, 1156 High Street, Santa Cruz, CA USA; 2 School of Mathematical and Statistical Sciences, Clemson University, 105 Sikes Hall, Clemson, NC USA; 3 School of Mathematical Sciences, Lancaster University, Bailrigg, Lancaster, UK; 4 Statistics Department, University of California Santa Cruz, 1156 High Street, Santa Cruz, CA USA; 5 Geophysical Fluid Dynamics Laboratory, NOAA, 201 Forrestal Road, Princeton, NJ USA; 6 Independent Researcher, Hamilton, NJ USA

**Keywords:** Atmospheric science, Climate-change impacts

## Abstract

With record-breaking temperatures over 2023-2024 and extreme events observed around the world, there is currently a debate in the scientific literature and media as to whether global warming is accelerating. Here we address this question from a regional perspective, determining regions where an increase in the rate of warming is detected in surface temperature observations. Our results show that there are coherent space-time systems of acceleration in warming and shift toward warming since 1970 that are consistent across multiple surface temperature datasets. We show that accelerated warming and warming onset have occurred in different regions and are mostly present at mid-latitudes of the Northern Hemisphere. There is notable variability in the onset of regional warming accelerations: the timings vary from the 1980s to 2010s. Our results provide some nuance on the ongoing debate about global warming acceleration by identifying the source regions experiencing changes in the rate of warming and their timings.

## Abstract (web summary)

Addressing the debate about global warming acceleration, this study identifies key regions that underwent recent warming shifts. Results show that regional warming speed-ups vary in timing from the 1980s to 2010s and occurred predominantly in the Northern mid-latitudes.

## Introduction

The years 2023 and 2024 were the two warmest recorded since observations of global surface temperature began in 1850^1^. These record-breaking temperatures, along with climate and weather events observed around the world, have spurred a debate as to whether global warming may be accelerating^2–6^.

An explanation for the warming spike relates to El Niño Southern Oscillation (ENSO). In 2023, there was a switch from a long La Niña to El Niño regime. The transition in ENSO conditions can explain, in part, warming spikes such as those observed in 2023 and 2024 in the context of a long-term anthropogenic warming trend^7–15^. However, the magnitude of the 2023 anomaly has prompted debate about whether ENSO alone is a sufficient explanation. While Raghuraman et al. ^7^, find that the La Niña–El Niño transition is sufficient to explain the annual-mean warming spike, Rantanen and Laaksonen ^16^, argue that the extreme monthly anomaly observed in September 2023 is extremely unlikely to arise from internal variability and greenhouse gas forcing alone, suggesting a possible role for additional external forcings such as the Hunga Tonga volcanic eruption or a record-low planetary albedo ^17^.

Another explanation for the exceptional temperatures observed in 2023–2024 involves an acceleration in the rate of global warming after 2010 due to an increase in Earth’s energy imbalance from reductions in cooling aerosols^4,5,18,19^. In particular, the 2020 International Maritime Organization (IMO) regulation limiting sulfur content in shipping fuel led to a rapid reduction in ship-track aerosols, representing an abrupt decrease in anthropogenic cooling that may have contributed specifically to the 2023–2024 warming spike^20^. During this time, the overall effective anthropogenic radiative forcing has increased, which may have been expressed as a recent acceleration in surface temperature, although with large uncertainties^21^. However, when analyzing observed surface temperatures, an acceleration in warming circa 2010 is not yet detectable on the global scale^2,6,22^. Additional years of observations are necessary to distinguish a change in the rate of global warming from natural variability^2^. Indeed, caution is necessary when interpreting changes in the rate of warming over short periods of time, as illustrated by the previous debate on the alleged pause in global warming from about 1998–2015^23–27^. Internal climate variability, as simulated by a climate model, can lead to decadal or even multi-decadal periods of global mean warming accelerations and warming pauses, when superimposed on a background anthropogenic global warming signal ^28^.

Although many timely publications provide important information on the timing of a recent potential acceleration in global warming, a data mining exercise is implemented here to uncover regions that may have undergone accelerations and to identify when they occurred. The response of the Earth’s climate system to anthropogenic forcings is not uniform in space or time ^29^. Land warms faster than the ocean even at equilibrium, driven primarily by differences in heat capacity, evaporative cooling, atmospheric moisture transport, and lapse-rate feedbacks ^30,31^. Beyond this land/ocean contrast, the rate of surface warming can vary considerably in space: ocean/atmosphere circulation may keep some regions cooler or warmer than average, changes in albedo, sea ice, and meltwater at high latitudes can impact warming at local and larger scales, and radiative forcing varies regionally due to differences in aerosol emissions and the distributions of trace gases ^29,32–35^. Similarly, the rate of surface warming fluctuates on interannual to multidecadal timescales (though presumably shorter timescales than the century-scale greenhouse gases response) due to natural variability and changes in external forcings such as volcanic eruptions, solar radiation and aerosol emissions ^29,36,37^. The detection of consistent regional changes in observed temperature can better focus and inform mitigation/adaptation efforts, as well as shed light on the potential mechanisms driving these changes in surface temperature. We contribute to this effort by determining space-time patterns of the accelerated rate of observed surface warming since 1970 through a systematic analysis of multiple gridded datasets. We chose 1970 as our start date because it marks the well-documented global warming acceleration driven by a rapid change in global radiative forcing^38^. This period focuses on recent temperature trends while avoiding increased data uncertainty introduced by earlier data biases and sparsity.

We utilize changepoint techniques to detect when and where there has been a warming acceleration since the 1970s. These types of models can identify changes in time series trends and have been used to interpret surface temperature records^2,24,27,39,40^. Five gridded surface temperature datasets are analyzed at the annual time scale (see “Methods”) over 1970–2024. Our detection results assume that natural variability can be captured in observations by a first-order autocorrelation process. This represents a short-term memory that decays exponentially over time, although long-term memory models that decay as a power law could also be used in a future study employing longer available data records^41–43^. Our models also assume that changes in the rate of warming, such as accelerations/decelerations, can be characterized by piecewise-linear models. Our results show that, on a regional scale, there are coherent regions showing a signal of warming acceleration since 1970 across all datasets.

## Results

**Fig. 1:**
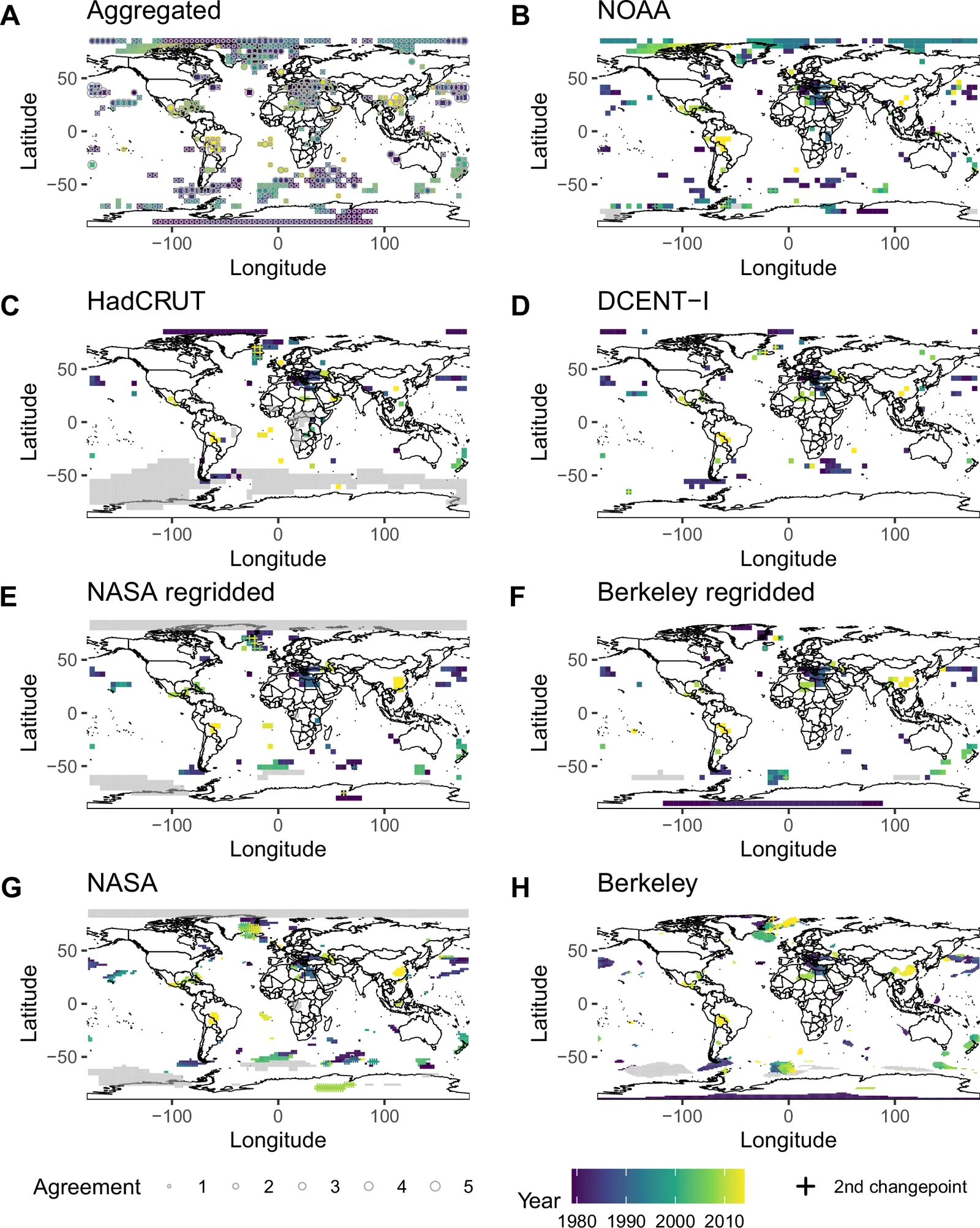
Timings of regional changes in the rate of warming detected since 1970 in multiple datasets obtained using a Bayesian Information Criterion (BIC) penalty. Panels show different datasets analyzed (**A**) Aggregated, **B** NOAA, **C** HadCRUT, **D** DCENT, **E** NASA regridded, **F** Berkeley regridded, **G** NASA and (**H**) Berkeley. To obtain the aggregated results, the Berkeley and NASA datasets were regridded to a 5^∘^ × 5^∘^ resolution and were analyzed with changepoint models. The average timing of change across datasets (if a change is detected) is shown. The size of the circles indicate the agreement across datasets in panels B-F in 5^∘^ × 5^∘^ resolution, small circle- low agreement, large circle- high agreement. White areas indicate where changes are not detectable and gray areas indicate missing data. When more than one change is detected, a cross symbol is superposed to the map to indicate the timing of the second change.

**Fig. 2:**
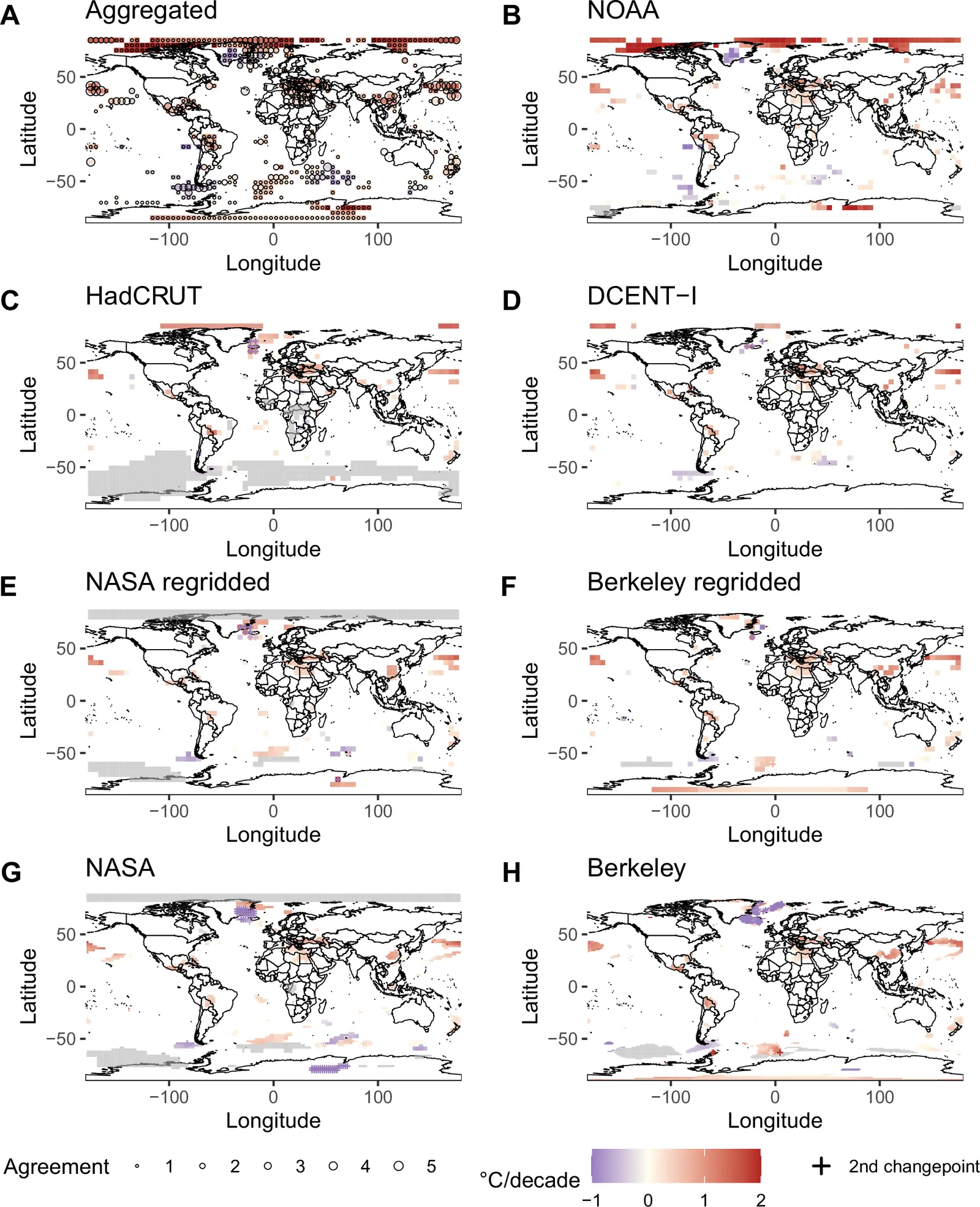
Magnitudes of regional changes in the rate of warming detected since 1970 in multiple datasets obtained using a Bayesian Information Criterion (BIC) penalty. Magnitudes are expressed as differences in slopes after and before changepoint. Panels show different datasets analyzed (**A**) Aggregated, **B** NOAA, **C** HadCRUT, **D** DCENT, **E** NASA regridded, **F** Berkeley regridded, **G** NASA and (**H**) Berkeley. To obtain the aggregated results, the Berkeley and NASA datasets were regridded to a 5^∘^ × 5^∘^ resolution and were analyzed with changepoint models. The average magnitude of change across datasets (if a change is detected) is shown. The size of the circles indicate the agreement across datasets shown in panels B-F in 5^∘^ × 5^∘^ resolution, small circle- low agreement, large circle- high agreement. White areas indicate where changes are not detectable and gray areas indicate missing data. When more than one change is detected, a cross symbol is superposed to the map to indicate the magnitude of the second change.

**Fig. 3:**
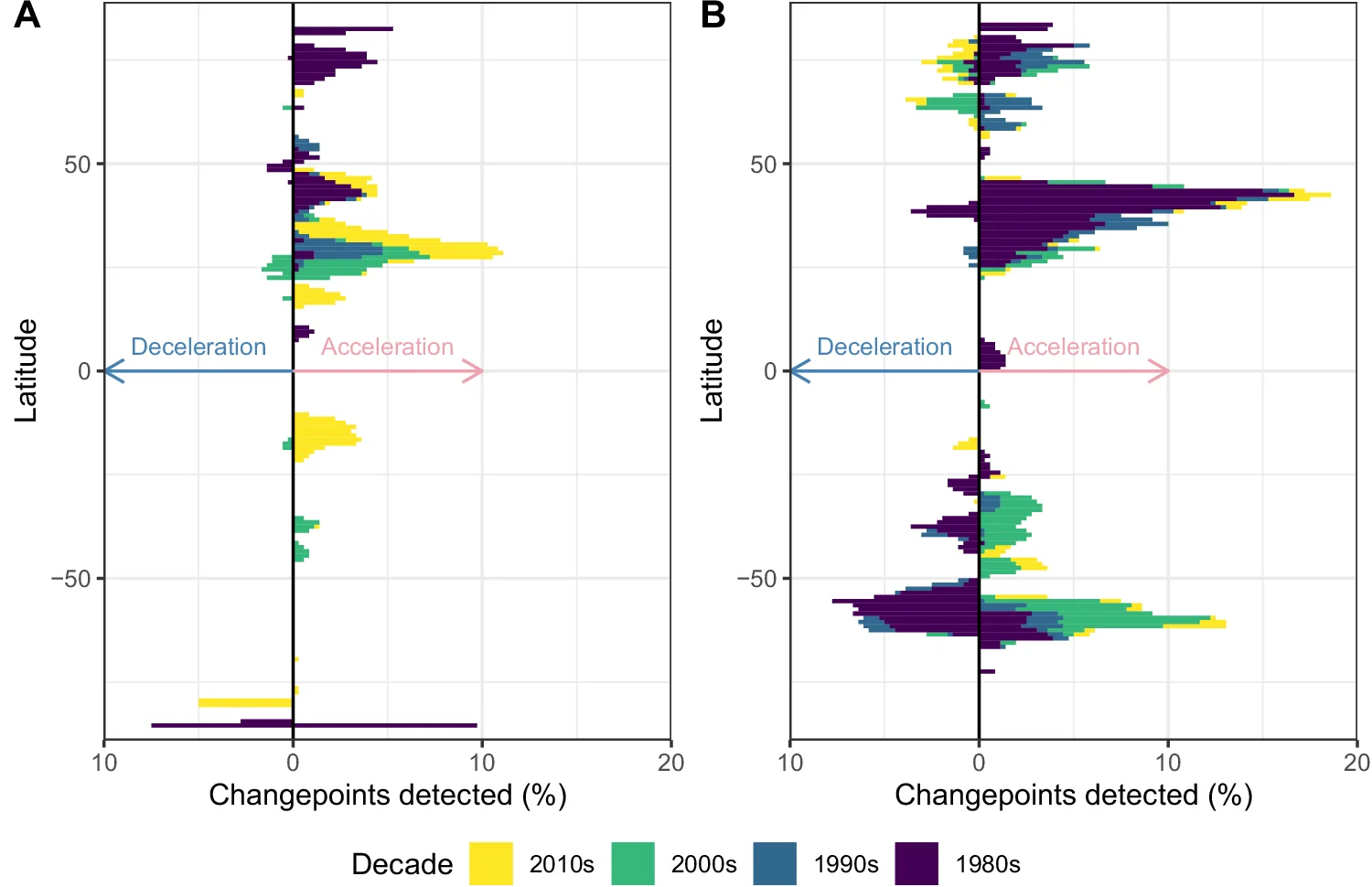
Percentage of changepoints detected by latitude based on the Berkeley dataset. Changepoints are presented separately for (**A**) land grids and (**B**) ocean grids. The double horizontal axis separates changes identified as accelerations (and shifts toward warming) vs decelerations (and shifts toward cooling), and the color represents the decade of change.

Piecewise linear models are fitted to annual surface temperature time series over 1970–2024 for all grid cells in all surface temperature datasets (see “Methods”). Between 4–10% of grid cells have experienced at least one detectable change in the rate of warming across all datasets (Table 1). The timings of detected changepoints are presented in Fig. 1. The datasets analyzed here vary in spatial resolution and are thus difficult to compare directly. As such, we have regridded the finer resolution datasets (Berkeley and NASA) to a coarser resolution by averaging data on a 5^∘^ × 5^∘^ grid and re-analyzed. Fig. 1A presents the aggregated average start year of changes in the rate of warming obtained when analyzing all datasets on a 5^∘^ × 5^∘^ grid resolution (Fig. 1B–F). Results obtained from higher resolution datasets are also presented in Fig. 1H–G. There are clearly space-time systems of changes in the rate of warming since 1970: the timings and locations of those changes are mostly consistent across datasets. Figure 2 presents the magnitude of the changes detected in all datasets. The regions that have experienced a change in the rate of warming since 1970 show predominantly accelerations, or transition from cooling or near-zero trends to warming (Fig. 3).

**Table 1:** Number of changepoints detected in each dataset across all grid cells(%)

	Number of changepoints (%)	
Dataset	1	2
NOAA	9.6	0.2
HadCRUT	4.3	0.2
DCENT-I	4.1	0.2
NASA regridded	4.6	0.3
Berkeley regridded	5.6	0.1
NASA	4.6	0.5
Berkeley	5.7	0.2

The maximum number of changepoints detected across all datasets is two.

Although some of the space-time systems highlighted here experienced substantial accelerated warming, they account for at most 23% of grid cells per latitude band and are mostly located at mid-latitudes in the Northern hemisphere (Fig. 2). Timings of changes in the rate of warming vary depending on whether they occur over land or ocean (Fig. 3). Over land, most changes in the rate of warming represent an acceleration and take place in the Northern hemisphere in all decades (Fig. 3A). Over the ocean, warming accelerations and shifts toward warming are predominant in the Northern hemisphere, whilst the Southern hemisphere has experienced both warming slowdowns and accelerations in similar proportions (Fig. 3B). Slowdowns in warming occurred mostly in the Southern Ocean and in the 1980s. These results are consistent with previous analyses performed on the global scale showing that an acceleration in global warming post-1970 is not yet detectable in global surface temperatures^2^. We extended this analysis until 2024 here (see Supplementary Information; Fig. S1).

**Fig. 4:**
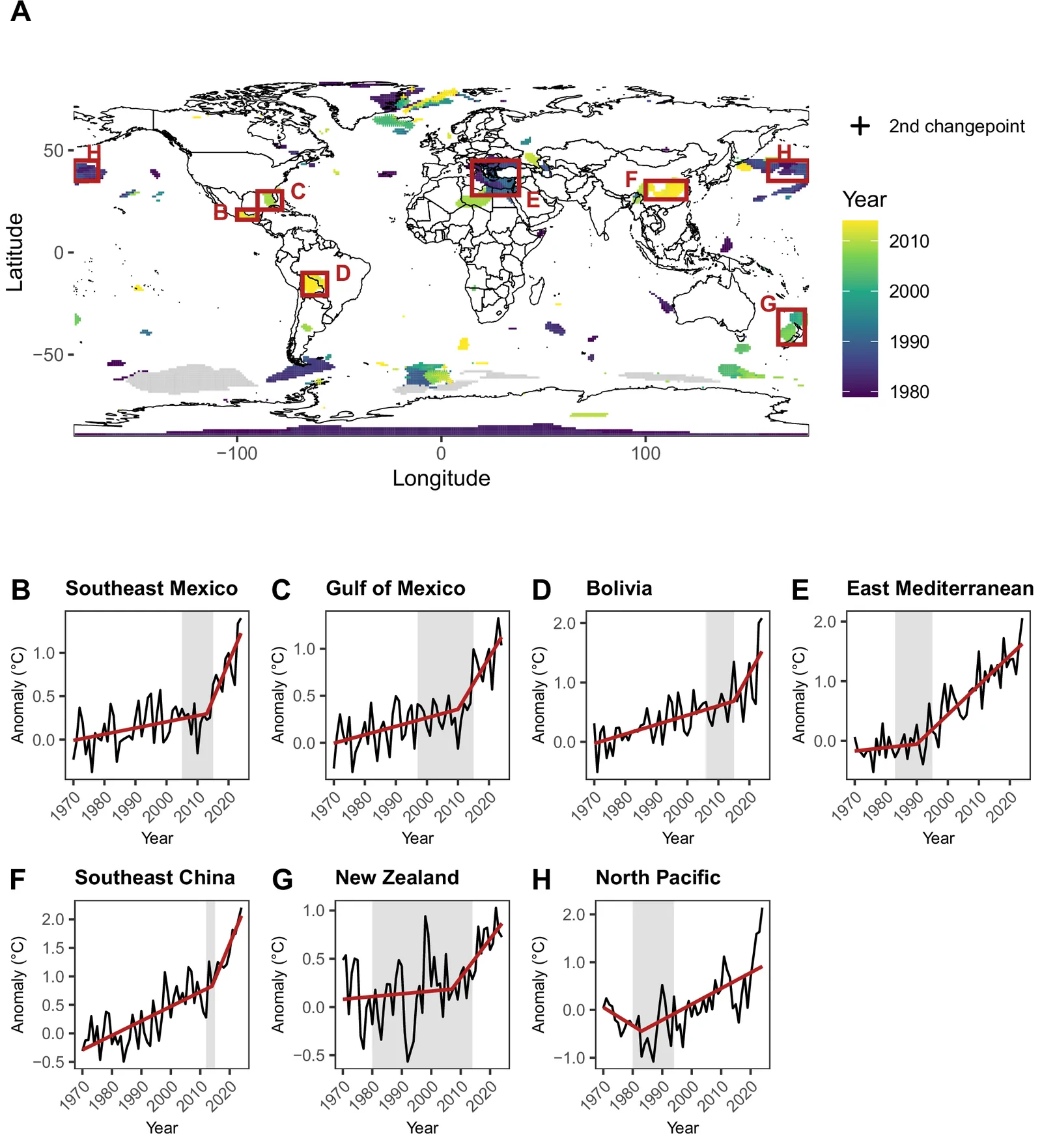
Regional averages for areas showing widespread changes in the rate of warming for the Berkeley dataset. **A** Timing of changes detected (same as Fig. 1H) with superposed areas definitions. **B**–**H** Time series average of surface temperature in the corresponding box with superposed model fit (red). Gray areas indicate 95% confidence intervals for changepoint timings.

Figure 4A focuses on regions that have experienced accelerations in warming or shifts toward warming since 1970 in most data sets, as identified in Fig. 1A. Widespread changes in the rate of warming emerge in southeast Mexico and the Gulf of Mexico, Bolivia, and southeast China in the 2010s (although the change in southeast China is present in all but the HadCRUT dataset and the change in Bolivia is present in all but the DCENT-I dataset) (Fig. 1). In the 2000s, an acceleration in warming in and along the coast of New Zealand is detected. In the 1980s and 1990s, widespread changes in the rate of warming were observed in the eastern Mediterranean area and in the North Pacific. We focus on regions between 60N and 60S where data coverage over land and in the ocean is more complete ^44,45^. The seven regions with changes in trends identified in Fig. 4A were detected in most datasets, representing agreement across the surface temperature observations. There are also uncertainties coming from processes generating the individual datasets. To quantify that uncertainty, we have analyzed the individual ensemble members from the DCENT-I and HadCRUT datasets (see Supplementary Information; Figs. S2–S3). These results show that the seven regions identified correspond to large agreement across ensemble members, except Bolivia for the DCENT-I dataset (Fig. S2). In the HadCRUT ensemble, most regions identified also show strong agreement across ensemble members except for southeast Mexico, the Gulf of Mexico and southeast China (Fig. S3).

**Fig. 5:**
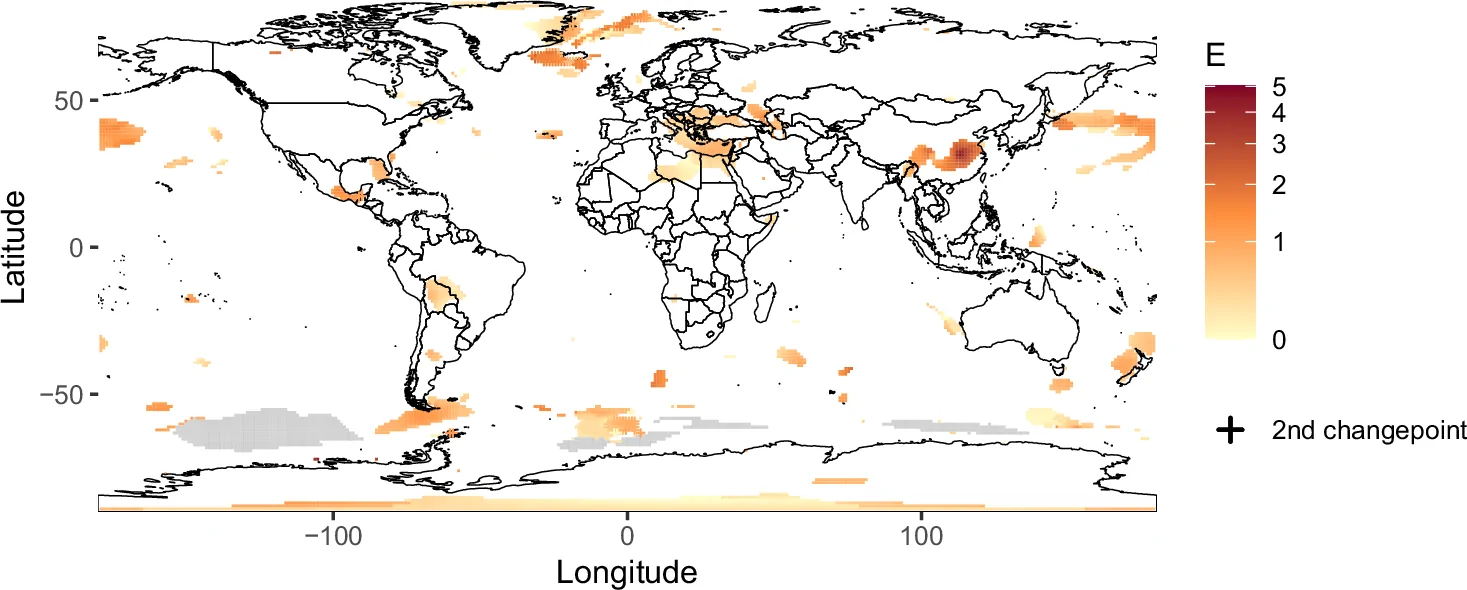
Changepoint energy (E) for grid cells where a change was identified, based on the Berkeley dataset. White areas indicate where changes are not detectable, and gray areas indicate missing data. When more than one change is detected, a cross symbol is superposed on the map to indicate the energy of the second change.

Figure 4B–H presents the average time series for the regions that have experienced accelerations in warming or shifts toward warming since 1970 based on the Berkeley dataset. Regional averages are calculated first by computing the average within the corresponding box in Fig. 4A, then the time series is analyzed. Uncertainties in the changepoint timing captured by 95% confidence intervals (conditional on the presence of one change) are illustrated in Fig. 4B–H, and available at a grid cell level in Fig. S4. Table 2 presents the magnitude and timings of these changes. In some regions, the acceleration in warming detected is steep. For example, the Southeast Mexico area shows approximately a tenfold increase in the rate of warming after 2012 and Southeast China on the order of a fivefold increase after 2013. Some changes are more easily identified than others depending on their energy (E), which involves the timing of the change, the magnitude of the change and the background noise or variability (see “Methods”). Figure 5 shows the changepoint energy for the grid cells with detected changes in the Berkeley dataset and the energy computed on regional averages in Fig. 4 are presented in Table 2.

**Table 2:** Timings, magnitudes and changepoint energy (E) of regional accelerations detected (estimated using the Berkeley dataset)

Region	Timing	Trend	Trend	E
	(95% CI)	before	after	
Southeast Mexico	2012	0.007	0.084	1.24
	(2004–2014)	(0.002–0.012)	(0.066–0.103)	
Gulf of Mexico	2009	0.009	0.055	0.67
	(1996–2014)	(0.004–0.014)	(0.040–0.070)	
Bolivia	2014	0.016	0.093	0.35
	(2005–2014)	(0.011–0.021)	(0.028–0.158)	
East Mediterranean	1989	0.006	0.050	0.49
	(1982–1994)	(−0.003–0.015)	(0.045–0.054)	
Southeast China	2013	0.025	0.123	2.57
	(2011–2014)	(0.016–0.034)	(0.104–0.143)	
New Zealand	2006	0.003	0.040	0.87
	(1979–2013)	(−0.014–0.019)	(0.033–0.047)	
North Pacific	1982	-0.038	0.033	0.87
	(1979–1993)	(−0.088–0.011)	(0.022–0.045)	

Confidence intervals (95% CI) on changepoint timings and trends (in ^∘^C/year) before and after the changepoint are presented below the estimates inside parentheses. The confidence intervals are conditional on one changepoint.

When considering the changepoint energy, the largest change in the rate of warming is an acceleration in Southeast China with SNR = 2.6. As an example, we will take the average Southeast China time series shown in Fig. 4F. A changepoint was detected at *τ*_1_ = 44, which corresponds to the year 2014. The trends before and after the changepoint are *β*_1_ = 0.025 and *β*_2_ = 0.123, and the standard deviations are *σ*_1_ = 0.315 and *σ*_2_ = 0.129. Plugging these values in equation (7) yields a *E* of 2.6. Both changes in trend and variance contribute to a large energy here.

The results discussed above were obtained using the Bayesian Information Criterion (BIC) penalty for model selection, and highly depend on this choice of model penalty. The BIC penalty has been proven to perform best in terms of Type I error and detection power with the types of models fitted here^46^. However, as a measure of sensitivity, we repeated our analysis with a relaxed penalty that does not account for the changepoint timing in the penalty term (see Supplementary Information). This makes changepoints easier to detect and increases the number of grid cells showing a change in the rate of warming. Consequently, under a relaxed penalty there is an expansion in the regions for which the rate of warming changes (see Supplementary Information; Figure S5). Although the proportion of changes identified increases with a relaxed penalty, one must be cautious in interpreting these changes as the rate of false detections also increases with a smaller penalty (see Supplementary Information; Table S1). The method performance in detecting changes is further assessed in Supplementary Information Figure S6. As expected, the detection power increases as the changepoint energy increases. Changes that took place in the middle of the time series (e.g., in the 1990s and 2000s) are also easier to detect than changes that occurred early or late in the time series as there is a more balanced number of data points prior and after the change (Supplementary Information; Figure S6).

An alternative approach to characterize a potential acceleration in warming is through a quadratic trend component in a regression model^22^. We consider it here as a complementary approach to assess the presence of accelerations in surface warming (Supplementary Information; Fig. S7). Regions with a significant quadratic trend include those identified using piecewise linear models in Figs. 1-2. As such, our results appear robust to the choice of definition for an acceleration.

## Discussion

The presence of space-time systems of changes in the rate of warming since 1970 was determined by changepoint detection. We show that since 1970, there have been more accelerations (and shifts toward warming) than decelerations (and shifts toward cooling) in surface temperature trends. We expose regions that have experienced an acceleration (and shift toward warming) in temperature trends with timings varying from the 1980s to the 2010s. The regions identified only comprise a small portion (less than 10%) of the grid cells analyzed. The variability in trend changes appears to be greater over the ocean, where both accelerations (and shifts toward warming), as well as decelerations (and shifts toward cooling) occur, whereas land warming changes are more exclusively accelerating. Warming slowdowns were mainly detected over the ocean in the Southern hemisphere. Warming accelerations across land and ocean are predominantly in the Northern hemisphere at midlatitudes. Regional changes identified that are consistent across datasets are: the Gulf of Mexico and southeast Mexico, eastern Mediterranean, southeast China, Bolivia, the North Pacific and New Zealand.

Our findings are based on piecewise linear models to represent changes in the rate of warming. To assess the level of sensitivity to a different definition for changes in the rate of warming, we analyzed surface temperature data with a quadratic trend model (Supplementary Information; Fig. S4). The quadratic trend analysis is consistent with the changepoint analysis across most regions, but instead fits a smooth continuous acceleration or deceleration over the full record. Similarly, the penalty used has some impact on changes detected, which we assessed in the Supplementary Information. Furthermore, our results depend on the assumption that the noise in surface temperatures behaves as a first-order autocorrelation, which represents a short-memory that decays exponentially over years. Other types of models used to describe the noise in surface temperature observations include a long-memory model where the decay is a power law^47–49^. We do not consider these models here as identifying long-memory requires longer time series ^42,43^. However, we note that, long-memory is more predominant in the oceans than over land ^43,49^. As such, changes identified in sea surface temperatures (e.g., the North Pacific) should be taken with caution. Finally, we note that we treated each grid cell independently of each other here despite the presence of spatial correlations. A space-time model could potentially improve detection through borrowing power from neighboring grid cells and should be the subject of a future study.

Advancements in climate science rely on integrating multiple methodologies, including fundamental physical principles, observational data analytics, and computational modeling. Here, we use statistical analysis of observations to provide some nuance to the ongoing debate about an acceleration in global warming. Future studies should confront model simulations with results obtained here based on observations to identify any discrepancies and conduct formal attribution studies^50^. While our focus is on surface temperatures, we acknowledge that there are other indicators of global heating (e.g., ocean heat content) that may show an acceleration that are not considered here^51^.

Our results are consistent with previous work suggesting that it is too early to detect an acceleration in surface warming at the global level^2^. The regions where we detect accelerations are limited, and their timings vary considerably. Alternatively, recent studies argue for an acceleration in global warming since 2010 due to reduction in aerosols^4,5^. While our results do not suggest widespread warming acceleration in 2010, we expose regions with accelerated warming at that time in the Gulf of Mexico, southeast Mexico, and southeast China. These results are potentially consistent with aerosol-driven changes, which are short-lived and regionally constrained. For example, Mexico has released a plan to reduce aerosol emissions by 2030^52^ and China’s sulfur dioxide (SO_2_) emissions from coal-fired power plants and industry have declined since 2007^53^ and have been linked to accelerated warming in East Asia and in the Pacific^54^. The warming acceleration detected in the late 1980s in the eastern Mediterranean region is consistent with reductions in aerosols in that region and brightening after the 1990s^55,56^. However, further scrutiny is necessary to attribute the causes of the changes observed here and should be the subject of upcoming studies. Changes in aerosols in different regions may have diverse and even opposite impact on the Earth’s energy budget^57^.

While these results point to aerosol reductions as a potential driver of the detected warming accelerations, we acknowledge that modes of internal variability could also contribute to the observed spatial patterns. Low-frequency variability phenomena such as the Pacific Decadal Oscillation (PDO) and the Atlantic Multidecadal Variability (AMV) operate on multi-decadal timescales and have well-characterized spatial fingerprints that could project onto the regional patterns of changepoints detected here. For instance, a phase shift in the PDO towards its positive phase is associated with enhanced warming over the North Pacific and parts of the western Americas ^58^, which could reinforce or confound aerosol-driven signals in those regions. Similarly, AMV could contribute to the detected changes over the North Atlantic and surrounding land areas ^59^. As for regions with decelerations in temperature detected in the Southern Ocean in the 1980s, these changes have been documented and attributed to increased wind-driven northward sea-ice transport that strengthened surface stratification and reduced upward heat flux ^60^, as well as freshwater forcing from melting Antarctic ice sheets ^61^. Disentangling the relative contributions of internal variability and external forcing to the detected changepoints is beyond the scope of the present study.

It should be noted that changes identified in gridded surface temperatures through this data mining exercise could be artificial and due to modifications in measurement procedures. However, all data sets used here were independently corrected for data inhomogeneities, which reduces the risk of artificial changes, but does not eliminate it. Similarly, the data in areas of sea ice are treated differently across datasets, which may affect our results at high latitudes ^62^. The five datasets used here all blend land surface air temperature with sea surface temperature, but differ in their treatment of sea ice regions. For example, NASA and HadCRUT use surface air temperature anomalies over ice-covered areas, while Berkeley uses SST measurements directly rather than as air temperature proxies ^63–65^. A caveat to our analysis is that the set of land-ocean combined surface temperature datasets we examine here does not represent the full range and variation of regional and area-averaged SST trends seen across different SST datasets ^62^. That is, the combined land-ocean datasets we examined tend to show much SST trend behavior that is more similar and consistent across the datasets than is seen across a wider sampling of SST datasets.

Monitoring regional changes in the rate of warming is important for local populations to develop climate adaptation strategies. Regions with large populations and low socioeconomic development are especially vulnerable to accelerated warming^66^. Similarly, ecosystems are more affected by rapid climate changes than gradual changes, so accelerated warming would be expected to increase the impacts of climate change on ecosystems relative to what would occur for a more constant gradual warming rate.

## Methods

### Data

The following five gridded datasets of surface temperatures were analyzed in this study:

- The Berkeley Earth Surface Temperatures (Berkeley) gridded dataset^64,67^. Monthly anomalies cover the period 1850–2024 on a 1^∘^ × 1^∘^ grid.
- The Goddard Institute for Space Studies Surface Temperature Analysis (GISTEMPv4) at the National Aeronautics and Space Administration (NASA) ^68,69^. Gridded monthly surface temperatures span 1880–2024 on a 2^∘^ × 2^∘^ grid.
- The Merged Land-Ocean Surface Temperature Analysis from the National Oceanic and Atmospheric Administration (NOAAGlobalTemp v6.1)^70,71^. Gridded monthly surface temperature data are available from 1850–2024 on a 5^∘^ × 5^∘^ grid.
- The Hadley Center/Climatic Research Unit, Version 5 (HadCRUT), surface temperature ^65,72^. Gridded monthly surface temperature anomalies from 1850–2024 on a 5^∘^ × 5^∘^ grid.
- Globally infilled extension of the Dynamically Consistent ENsemble of Temperature (DCENT-I)^73–75^. Gridded monthly surface temperature anomalies from 1850–2024 on a 5^∘^ × 5^∘^ grid.

For all datasets, we compute annual mean surface temperature anomalies time series (i.e., evenly spaced data in time) over the period 1970–2024 to focus on changes in the rate of warming since 1970. We restrict our analysis to grid cells with complete, uninterrupted observational time series (i.e., grid cells containing missing values are excluded prior to model fitting).

### Statistical analysis

We analyze gridded surface temperature time series using changepoint detection to determine whether changes in the trend since 1970 are present. The methodology builds upon^2^ and references therein.

We model the surface temperature time series, *X*_*t*_, *t* = 1, …, *N*, at each grid cell independently as

1 $${X}_{t}=E[{X}_{t}]+{\epsilon }_{t},$$

where *E*[*X*_*t*_] is a piecewise linear regression function that characterizes the expectation and *ϵ*_*t*_ represents the model errors. The regression function with *m* changepoints at times *τ*_1_, …, *τ*_*m*_ (1 < *τ*_1_ < ⋯ < *τ*_*m*_ < *N*) partitions the series into *m* + 1 segments:

2 $$E[{X}_{t}]=\left\{\begin{array}{cc}{\alpha }_{1}+{\beta }_{1}t,& 1\le t\le {\tau }_{1},\\ {\alpha }_{2}+{\beta }_{2}t,& {\tau }_{1} < t\le {\tau }_{2},\\ \vdots &\vdots \\ {\alpha }_{m+1}+{\beta }_{m+1}t,& {\tau }_{m} < t\le N,\\ \end{array}\right.$$

where *β*_*i*_ denotes the annual slope in segment *i*. We enforce a continuous join constraint across segments^27^:

3 $${\alpha }_{i}+{\beta }_{i}{\tau }_{i}={\alpha }_{i+1}+{\beta }_{i+1}{\tau }_{i},\qquad 1\le i\le m.$$

The errors {*ϵ*_*t*_} follow a segment-specific first-order autocorrelation (AR(1)) process

4 $${\epsilon }_{t}={\phi }_{i}{\epsilon }_{t-1}+{Z}_{t},\qquad t\in \,{\mbox{segment}}\,i,$$

where {*Z*_*t*_} is independent and identically distributed Gaussian white noise with mean zero and variance ${\sigma }_{i}^{2}$, and the first-order autocorrelation, *ϕ*_*i*_, varies in ( − 1, 1). The parameters of the error process are allowed to vary across the different climate regimes ^2,24^.

Model estimation minimizes the penalized negative log-likelihood objective function:

5 $$O(m;{\tau }_{1},\ldots,{\tau }_{m})=-2\ln (L(m;{\tau }_{1},\ldots,{\tau }_{m}))+P(m),$$

where the likelihood is denoted by *L*(*m*; *τ*_1_, …, *τ*_*m*_) and is penalized by the penalty function, *P*(*m*). The likelihood is evaluated conditionally and segment-by-segment with AR(1) parameters (*ϕ*_*i*_) estimated via Maximum Likelihood Estimation (MLE), allowing both the autoregressive parameters and the residual variance to vary locally across changepoints.

The larger the penalty function, the fewer changepoints will be detected by the model. Specifically, we use the Bayesian Information Criterion (BIC) penalty here:

6 P(m)=(4m+4)ln(N),

accounting for 4*m* + 4 free parameters (${\alpha }_{i},{\beta }_{i},{\phi }_{i},{\sigma }_{i}^{2}$ plus *m* changepoint locations) in the model. An alternative representation that does not include the changepoint timings in the number of parameters is included in Supplementary Information.

Global optimization over all valid segmentations is guaranteed using the Pruned Exact Linear Time (PELT) dynamic programming algorithm ^76^, subject to a minimum segment length constraint of 10 years. In this framework, the selected model is treated as the best-fitting description of the data. We apply the above changepoint detection to each grid cell independently, acknowledging that spatial dependence across grid cells is present.

To evaluate detection difficulty, we compute the changepoint energy *E*, adapted from^77^. For a single changepoint model, (*E*) is defined as:

7 $$E=\left| \frac{{\beta }_{2}\cdot T}{{\sigma }_{2}}-\frac{{\beta }_{1}\cdot T}{{\sigma }_{1}}\right| \sqrt{\frac{({\tau }_{1}-1)(N-{\tau }_{1})}{N}},$$

where *σ*_1_ and *σ*_2_ represent the standard deviations of the process *X*_*t*_ over *t* ∈ [1, *τ*_1_] and *t* ∈ [*τ*_1_ + 1, *N*] respectively, and *T* = 1 year is a dimensionless scaling factor. The first term of Equation (7) considers the relative magnitude of the trend change relative to the variance in each segment, while the second term accounts for the timing effect. Detecting a change located in the middle of the time series is easier than towards the boundaries. Larger energy values indicate greater detectability.

For grid cells exhibiting a single change (*m* = 1), confidence sets for changepoint timing *τ*_1_ are constructed using the conditional likelihood ratio framework ^78,79^. We evaluate the joint negative log-likelihood given a candidate changepoint *τ*:

8 $${{\Lambda }}(\tau )=-2\ln {L}_{1}(1:\tau )-2\ln {L}_{2}((\tau+1):N).$$

The optimal timing minimizes Λ(*τ*). The 100(1 − *α*)% confidence set *C* consists of all candidate timings *τ* satisfying:

9 $$C=\{\tau :{{\Lambda }}(\tau )-{{\min }_{k}} \, {{\Lambda }}(k) < \eta \},$$

with threshold:

10 $$\eta=\frac{3}{2}+2\ln (2\cdot {n}_{j,\max })-2\ln (\alpha )-\ln (N),$$

where ${n}_{j,\max }$ is the maximum window length determined via a rolling minimum of segment lengths (*k* = 2).

Conditional on the identified changepoint timing *τ* (and *m* = 1), 95% confidence intervals for the trend slopes are computed using linear regression adapted for autoregressive AR(1) residual errors. For each segment *i* ∈ {1, 2}, the slope *β*_*i*_ is initially estimated via Ordinary Least Squares (OLS) on normalized time coordinates, and the resulting residuals are used to estimate the first-order autocorrelation coefficient (${\hat{\phi }}_{i}$). To account for temporal autocorrelation, the OLS trend slope standard error (${s}_{{\beta }_{i}}$) is inflated using the asymptotic variance correction multiplier:

11 $$\sqrt{(1+{\hat{\phi }}_{i})/(1-{\hat{\phi }}_{i})}.$$

Trend slope confidence limits for segment *i* are then constructed as:

12 $${\hat{\beta }}_{i}\pm \frac{{z}_{1-\alpha /2}\cdot {s}_{{\beta }_{i}}\sqrt{\frac{1+{\hat{\phi }}_{i}}{1-{\hat{\phi }}_{i}}}}{{n}_{{{{\rm{seg}}}},i}}$$

where *z*_1−*α*/2_ denotes the standard normal critical value, and *n*_seg,*i*_ represents the length of segment *i*.

## Supplementary information

Supplementary Information

Transparent Peer Review file

## Data Availability

All datasets used in this study are publicly available. Gridded monthly surface temperature data from the Hadley Center/Climatic research Unit (HadCRUT.5.1.0.0) are available at https://www.metoffice.gov.uk/hadobs/hadcrut5/data/HadCRUT.5.1.0.0/download.html. Gridded monthly surface temperature data from the Merged Land-Ocean Surface Temperature Analysis of the National Oceanic and Atmospheric Administration (NOAAGlobalTemp v6.1.) are available at https://downloads.psl.noaa.gov/Datasets/noaaglobaltemp/v6.1/. The Berkeley Earth Surface Temperatures gridded data are available at (http://berkeleyearth.org/data). The Goddard Institute for Space Studies (GISS) Surface Temperature Analysis (GISTEMP) Gridded Monthly Temperature Anomaly Data are available at (https://data.giss.nasa.gov/gistemp/). The infilled Dynamically Consistent ENsemble of Temperature (DCENT-I) data are available at (https://dcent-i.github.io/). The processed gridded surface temperature data used in this study and results generated to produce figures are available at (https://doi.org/10.5281/zenodo.22018973^80^). These data include the number, year, magnitude, and energy of detected changepoints, and the processed datasets used for the analyses and figures presented in this paper.

## Code availability

Codes used to conduct the analyses and produce figures are available at (https://doi.org/10.5281/zenodo.19143048^81^.).

## References

[1] NOAA National Centers for Environmental Information: Monthly Global Climate Report for Annual 2024. https://www.ncei.noaa.gov/access/monitoring/monthly-report/global/202413 (2025).

[2] Beaulieu, C., Gallagher, C., Killick, R., Lund, R. & Shi, X. A recent surge in global warming is not detectable yet. *Commun. Earth Environ.* **5**, 576 10.1038/s43247-024-01711-1 (2024).

[3] Foster, G. & Rahmstorf, S. Global warming has accelerated significantly. *Geophys. Res. Lett.* **53**, 2025–118804 10.1029/2025GL118804 (2026).

[4] Hansen, J. E. et al. Global warming in the pipeline. *Oxf. Open Clim. Change* **3**, 008 10.1093/oxfclm/kgad008 (2023).

[5] Hansen, J. E. et al. Global warming has accelerated: Are the United Nations and the public well-informed? *Environ.: Sci. Policy Sustain. Dev.* **67**, 6–44 10.1080/00139157.2025.2434494 (2025).

[6] Samset, B. H., Zhou, C., Fuglestvedt, J. S., Lund, M. T., Marotzke, J. & Zelinka, M. D. Steady global surface warming from 1973 to 2022 but increased warming rate after 1990. *Commun. Earth Environ.* **4**, 400 10.1038/s43247-023-01061-4 (2023).

[7] Raghuraman, S. P., Soden, B., Clement, A., Vecchi, G., Menemenlis, S. & Yang, W. The 2023 global warming spike was driven by the El Niño Southern Oscillation. *Atmos. Chem. Phys.* **24**, 11275–11283 10.5194/acp-24-11275-2024 (2024).

[8] Blanchard-Wrigglesworth, E., Bilbao, R., Donohoe, A. & Materia, S. Record warmth of 2023 and 2024 was highly predictable and resulted from ENSO transition and Northern hemisphere absorbed shortwave anomalies. *Geophys. Res. Lett.* **52**, 2025–115614 10.1029/2025GL115614 (2025).

[9] Xie, S.-P., Miyamoto, A., Zhang, P., Kosaka, Y., Liang, Y., Lutsko, N.J. What made 2023 and 2024 the hottest years in a row? *npj Clim. Atmos. Sci.* **8**, 1 10.1038/s41612-025-01006-y (2025).

[10] Samset, B.H., Lund, M.T., Fuglestvedt, J.S., Wilcox, L.J. 2023 temperatures reflect steady global warming and internal sea surface temperature variability. *Commun. Earth Environ.* **5**, 1 10.1038/s43247-024-01637-8 (2024).

[11] Li, K., Zheng, F., Zhu, J. & Zeng, Q.-C. El Niño and the AMO sparked the astonishingly large margin of warming in the global mean surface temperature in 2023. *Adv. Atmos. Sci.* **41**, 1017–1022 10.1007/s00376-023-3371-4 (2024).

[12] Min, S.-K.: Human influence can explain the widespread exceptional warmth in 2023. *Commun. Earth Environ.* **5**, 1 10.1038/s43247-024-01391-x (2024).

[13] Gyuleva, G., Knutti, R. & Sippel, S. Combination of internal variability and forced response reconciles observed 2023–2024 warming. *Geophys. Res. Lett.* **52**, 2025–115270 10.1029/2025GL115270 (2025).

[14] Huang, B. et al. Record high sea surface temperatures in 2023. *Geophys. Res. Lett.* **51**, 2024–108369 10.1029/2024GL108369 (2024).

[15] Yukimoto, S., Kawai, H., Oshima, N. & Deushi, M. Emerging effective radiative forcing in the radiative imbalance since 2010. *Geophys. Res. Lett.* **53**, 2025–119913 10.1029/2025GL119913 (2026).

[16] Rantanen, M. & Laaksonen, A. The jump in global temperatures in September 2023 is extremely unlikely due to internal climate variability alone. *npj Clim. Atmos. Sci.* **7**, 34 10.1038/s41612-024-00582-9 (2024).

[17] Goessling, H. F., Rackow, T. & Jung, T. Recent global temperature surge intensified by record-low planetary albedo. *Science* **387**, 68–73, 10.1126/science.adq7280 (2025). 39636934

[18] Hodnebrog, Ø. et al. Recent reductions in aerosol emissions have increased Earth’s energy imbalance. Communications Earth & Environment **5**, 1 10.1038/s43247-024-01324-8 (2024).

[19] Merchant, C. J., Allan, R. P. & Embury, O. Quantifying the acceleration of multidecadal global sea surface warming driven by Earth’s energy imbalance. *Environ. Res. Lett.* **20**, 024037 10.1088/1748-9326/adaa8a (2025).

[20] Gettelman, A. et al. Has reducing ship emissions brought forward global warming? *Geophys. Res. Lett.* **51**, 2024–109077 10.1029/2024GL109077 (2024).

[21] Jenkins, S. et al. Is anthropogenic global warming accelerating? *J. Clim.* **35**, 7873–7890 10.1175/JCLI-D-22-0081.1 (2022).

[22] Richardson, M. T. Prospects for detecting accelerated global warming. *Geophys. Res. Lett.* **49**, 2021–095782 10.1029/2021GL095782 (2022).

[23] Risbey, J. S. et al. A fluctuation in surface temperature in historical context: reassessment and retrospective on the evidence. *Environ. Res. Lett.* **13** 10.1088/1748-9326/aaf342 (2018).

[24] Beaulieu, C. & Killick, R. Distinguishing trends and shifts from memory in climate data. *J. Clim.* **31**, 9519–9543 10.1175/JCLI-D-17-0863.1 (2018).

[25] Cahill, N., Rahmstorf, S. & Parnell, A. C. Change points of global temperature. *Environ. Res. Lett.* **10**, 084002 10.1088/1748-9326/10/8/084002 (2015).

[26] Rajaratnam, B., Romano, J., Tsiang, M. & Diffenbaugh, N. Debunking the climate hiatus. *Climatic Change* **133**, 129–140 10.1007/s10584-015-1495-y (2015).

[27] Rahmstorf, S., Foster, G. & Cahill, N. Global temperature evolution: recent trends and some pitfalls. *Environ. Res. Lett.* **12**, 054001 10.1088/1748-9326/aa6825 (2017).

[28] Knutson, T.R., Zhang, R., Horowitz, L.W.: Prospects for a prolonged slowdown in global warming in the early 21st century. *Nat. Commun*. **7** 10.1038/ncomms13676 (2016). PMC5141387 27901045

[29] Risbey, J. S., Grose, M. R., Monselesan, D. P., O’Kane, T. J. & Lewandowsky, S. Transient response of the global mean warming rate and its spatial variation. *Weather Clim. Extremes* **18**, 55–64 10.1016/j.wace.2017.11.002 (2017).

[30] Manabe, S., Stouffer, R. J., Spelman, M. J. & Bryan, K. Transient responses of a coupled ocean–atmosphere model to gradual changes of atmospheric CO_2_. Part I. Annual mean response. *J. Clim.* **4**, 785–818 (1991).

[31] Sutton, R.T., Dong, B., Gregory, J.M.: Land/sea warming ratio in response to climate change: IPCC AR4 model results and comparison with observations. Geophysical Research Letters **34**, 2 10.1029/2006GL028164 (2007).

[32] Hansen, J., Sato, M. & Ruedy, R. Perception of climate change. *Proc. Natl. Acad. Sci.USA* **109**, 2415–2423 10.1073/pnas.1205276109 (2012). PMC3443154 22869707

[33] Samset, B. H., Wilcox, L. J. & Allen, R. J. Broader research efforts and assessments needed to uncover the complex climate effects of regional changes in aerosol emissions. *PLOS Clim*10.1371/journal.pclm.0000508 (2024).

[34] Ramanathan, V. et al. Climate-chemical interactions and effects of changing atmospheric trace gases. *Rev. Geophys.* **25**, 1441–1482 10.1029/RG025i007p01441 (1987).

[35] Ramaswamy, V. et al. *Radiative forcing of climate change. In: Climate Change 2001: The Scientific Basis. Contribution of Working Group I to the Third Assessment Report of the Intergovernmental Panel on Climate Change*. **6**, 349–416 Cambridge University Press https://www.ipcc.ch/site/assets/uploads/2018/03/TAR-06.pdf (2001).

[36] Marotzke, J. & Forster, P. M. Forcing, feedback and internal variability in global temperature trends. *Nature* **517**, 565–570, 10.1038/nature14117 (2015). 25631444

[37] Easterling, D. R. & Wehner, M. F. Is the climate warming or cooling? *Geophys. Res. Lett.* **36**, 08706 10.1029/2009GL037810 (2009).

[38] Bindoff, N.L. et al. *Detection and attribution of climate change: from global to regional*. In: Stocker, T.F., et al. (eds.) *Climate Change 2013: The Physical Science Basis. Contribution of Working Group I to the Fifth Assessment Report of the Intergovernmental Panel on Climate Change.* 869–928. Cambridge University Press 10.1017/CBO9781107415324.022 (2013).

[39] Seidel, D.J., Lanzante, J.R. An assessment of three alternatives to linear trends for characterizing global atmospheric temperature changes. *J. Geophys. Res. Atmos.* **109**, D14 10.1029/2003JD004414 (2004).

[40] Mudelsee, M. Break function regression. * Eur. Phys. J. Spec. Top.* **174**, 49–63, 10.1140/epjst/e2009-01089-3 (2009).

[41] Mudelsee, M. et al. *Climate Time Series Analysis: Classical Statistical and Bootstrap Methods*, 2nd edn. *Atmospheric and Oceanographic Sciences Library*, **51** 10.1007/978-3-319-04450-7 (Springer, 2014).

[42] Poppick, A., Moyer, E. J. & Stein, M. L. Estimating trends in the global mean temperature record. *Adv. Stat. Climatol., Meteorol. Oceanogr.* **3**, 33–53, 10.5194/ascmo-3-33-2017 (2017).

[43] Beaulieu, C., Killick, R., Ireland, D. & Norwood, B. Considering long-memory when testing for changepoints in surface temperature: a classification approach based on the time-varying spectrum. *Environmetrics* **31**, 2568, 10.1002/env.2568 (2020).

[44] Deser, C. et al. National Center for Atmospheric Research Staff: The Climate Data Guide: ICOADS Surface Marine Weather Observations. https://climatedataguide.ucar.edu/climate-data/icoads-surface-marine-weather-observations (2023).

[45] Lawrimore, J.H. et al. An overview of the global historical climatology network monthly mean temperature data set, version 3. *J. Geophys. Res. Atmos*. **116**, D19 10.1029/2011JD016187 (2011).

[46] Shi, X., Gallagher, C., Lund, R., Killick, R.: A comparison of single and multiple changepoint techniques for time series data. *Comput. Stat. Data Analysis* **170** 10.1016/j.csda.2022.107433 (2022).

[47] Vyushin, D.I., Kushner, P.J., Zwiers, F.: Modeling and understanding persistence of climate variability. *J. Geophys. Res. Atmos.* **117**, D21 10.1029/2012JD018240 (2012).

[48] Franzke, C. Nonlinear trends, long-range dependence, and climate noise properties of surface temperature. *J. Clim.* **25**, 4172–4183 10.1175/JCLI-D-11-00293.1 (2012).

[49] Løvsletten, O. & Rypdal, M. Statistics of regional surface temperatures after 1900: long-range versus short-range dependence and significance of warming trends. *J. Clim.* **29**, 4057–4068 10.1175/JCLI-D-15-0437.1 (2016).

[50] Simpson, I. R. et al. Confronting Earth system model trends with observations. *Sci. Adv.* **11**, 8035, 10.1126/sciadv.adt8035 (2025). PMC11900882 40073142

[51] Minière, A., Schuckmann, K., Sallée, J.-B. & Vogt, L. Robust acceleration of Earth system heating observed over the past six decades. *Sci. Rep.* **13**, 22975 (2023). 38151491 10.1038/s41598-023-49353-1 PMC10752897

[52] INECC: Mexico: Integrated SLCP Strategy to Improve Air Quality and Reduce the Impact of Climate Change. https://www.ccacoalition.org/policy-database/mexico-integrated-slcp-strategy-improve-air-quality-and-reduce-impact-climate-change (2020).

[53] Li, C. et al. India is overtaking China as the world’s largest emitter of anthropogenic sulfur dioxide. *Sci. Rep*. **7**, 1 10.1038/s41598-017-14639-8 (2017). PMC5680191 29123116

[54] Samset, B.H. et al. East Asian aerosol cleanup has likely contributed to the recent acceleration in global warming. *Commun. Earth Environ*. **6**, 1 10.1038/s43247-025-02527-3 (2025).

[55] Urdiales-Flores, D. et al. Drivers of accelerated warming in Mediterranean climate-type regions. *npj Clim. Atmos. Sci.* **6**, 1 10.1038/s41612-023-00423-1 (2023).

[56] Wild, M.: Global dimming and brightening: a review. *J. Geophys. Res. Atmos.* **114**, D10 10.1029/2008JD011470 (2009).

[57] Miinalainen, T., Kokkola, H., Lehtinen, K. E. J. & Kühn, T. Comparing the radiative forcings of the anthropogenic aerosol emissions from Chile and Mexico. *J. Geophys. Res.: Atmos.* **126**, 2020–033364, 10.1029/2020JD033364 (2021).

[58] Newman, M. et al. The Pacific decadal oscillation, revisited. *J. Clim.* **29**, 4399–4427, 10.1175/JCLI-D-15-0508.1 (2016).

[59] Zhang, R. et al. A review of the role of the Atlantic meridional overturning circulation in Atlantic multidecadal variability and associated climate impacts. *Rev. Geophys.* **57**, 316–375, 10.1029/2019RG000644 (2019).

[60] Haumann, F. A., Gruber, N. & Münnich, M. Sea-ice induced southern ocean surface buoyancy fluxes. *AGU Adv.* **1**, 2019–000132, 10.1029/2019AV000132 (2020).

[61] Kaufman, Z., Wilson, E., Purich, A., Beadling, R. & Li, Y. The impact of underestimated southern ocean freshening on simulated historical sea surface temperature trends. *Geophys. Res. Lett.* **52**, 2024–112639, 10.1029/2024GL112639 (2025).

[62] Menemenlis, S., Vecchi, G. A., Yang, W., Fueglistaler, S. & Raghuraman, S. P. Consequential differences in satellite-era sea surface temperature trends across datasets. *Nat. Clim. Change* **15**, 897–903, 10.1038/s41558-025-02362-6 (2025).

[63] Hansen, J., Ruedy, R., Sato, M., Lo, K.: Global surface temperature change. *Rev. Geophys.* **48**, 4 10.1029/2010RG000345 (2010).

[64] Rohde, R. A. & Hausfather, Z. The Berkeley Earth land/ocean temperature record. *Earth Syst. Sci. Data* **12**, 3469–3479, 10.5194/essd-12-3469-2020 (2020).

[65] Morice, C. P. et al. An updated assessment of near-surface temperature change from 1850: The HadCRUT5 data set. *J. Geophys. Res.: Atmos.* **126**, 2019–032361, 10.1029/2019JD032361 (2021).

[66] Sengupta, A., King, A. D. & Ryan, R. G. Inequity in population exposure to accelerated warming. *Geophys. Res. Lett.* **51**, 2024–110644, 10.1029/2024GL110644 (2024).

[67] Berkeley Earth: Berkeley Earth surface temperatures. https://berkeleyearth.org/data/ (2025).

[68] Lenssen, N. et al. A NASA GISTEMPv4 observational uncertainty ensemble. *J. Geophys. Res.: Atmos.* **129**, 2023–040179, 10.1029/2023JD040179 (2024).

[69] GISTEMP team: GISS Surface Temperature Analysis (GISTEMP), version 4. NASA Goddard Institute for Space Studies. https://data.giss.nasa.gov/gistemp/ (2026).

[70] Zhang, H.-M., Huang, B., Lawrimore, J., Menne, M., Smith, T.M.: *NOAA Global Surface Temperature Dataset (NOAAGlobalTemp)*, Version 6.1. NOAA National Centers for Environmental Information. 10.25921/9qth-2p70 (2026).

[71] Huang, B., Yin, X., Menne, M. J., Vose, R. & Zhang, H.-M. Improvements to the land surface air temperature reconstruction in NOAAGlobalTemp: An artificial neural network approach. *Artif. Intell. Earth Syst.* **1**, 220032, 10.1175/AIES-D-22-0032.1 (2022).

[72] Met Office Hadley Centre and University of East Anglia Climatic Research Unit: HadCRUT5: Ensemble near-surface temperature anomaly grids and time series, version 5.1.0.0. https://www.metoffice.gov.uk/hadobs/hadcrut5/data/HadCRUT.5.1.0.0/download.html (2026).

[73] Chan, D., Gebbie, G., Huybers, P., Kent, E.C. A dynamically consistent ENsemble of temperature at the Earth surface since 1850 from the DCENT dataset. *Sci. Data* **11**, 1 10.1038/s41597-024-03742-x (2024). PMC11364547 39214994

[74] Chan, D., Gebbie, G., Huybers, P., Kent, E.: DCENT: Dynamically Consistent ENsemble of Temperature at the Earth surface. *Harvard Dataverse* 10.7910/DVN/NU4UGW (2024). PMC11364547 39214994

[75] Chan, D. et al. DCENT-I: A globally infilled extension of the dynamically consistent ENsemble of temperature dataset. *Geosci. Data J.* **13**, 70054 10.1002/gdj3.70054 (2026).

[76] Killick, R., Fearnhead, P. & Eckley, I. A. Optimal detection of changepoints with a linear computational cost. *J. Am. Stat. Assoc.* **107**, 1590–1598 (2012).

[77] Verzelen, N., Fromont, M., Lerasle, M. & Reynaud-Bouret, P. Optimal change-point detection and localization. *Ann. Stat.* **51**, 1586–1610 10.1214/22-EJS2048 (2023).

[78] Siegmund, D. Confidence sets in change-point problems. *Int. Stat. Rev.* **56**, 31–48 (1988).

[79] Fang, X., Li, J. & Siegmund, D. Segmentation and estimation of change-point models: False positive control and confidence regions. * Ann. Stat.* **48**, 1615–1647 10.1214/19-AOS1861 (2020).

[80] Beaulieu, C., Johnson, A., Killick, R., Lanzante, J., Knutson, T.: Processed data and results for: Space-time signatures of surface warming accelerations since 1970 [Dataset] 10.5281/zenodo.22018974 (2026).42773170

[81] Beaulieu, C., Johnson, A., Killick, R., Lanzante, J., Knutson, T.: claudiebeaulieu/RegionalWarming: Code to accompany Space-time signatures of surface warming accelerations since 1970 (Version v2.0.0) 10.5281/zenodo.19143048 (2026).42773170

