# Supplementary material for: Space-time signatures of surface warming accelerations since 1970
Source: Nat Commun. 2026 Sep 22;17:9742. doi: 10.1038/s41467-026-77514-z (PMC13598101; doi:10.1038/s41467-026-77514-z)
Supplement: Supplementary file 1 [file 41467_2026_77514_MOESM1_ESM.pdf]

Supplementary Information

"Space-time signatures of surface warming accelerations since 1970"

1 Supplementary Notes

1.1 Analysis of globally averaged data

We analyze global mean surface temperatures (GMST) over the full record (Figure S1). An acceleration post 1970 is not detected, a result similar to Beaulieu et al. (2024) in which data was analyzed until 2023. We note that the results remain the same with the reduced penalty described in Equation 2 below. We also note that the results remain the same if the year 2025 is included in the analysis. The following GMST datasets were included here:

- The Hadley Centre/Climatic Research Unit, Version 5 (HadCRUT), surface temperature (Morice et al., 2021). This series is available at `https://www.metoffice.gov.uk/hadobs/hadcrut5/data/HadCRUT.5.1.0.0/download.html`.
- The Merged Land–Ocean Surface Temperature Analysis from the National Oceanic and Atmospheric Administration (NOAAGlobalTemp v6) of Vose et al. (2021). This series is available at `https://www.ncei.noaa.gov/data/noaa-global-surface-temperature/v6.1/access/timeseries/`.
- The Berkeley Earth Surface Temperatures (Berkeley) of Rohde and Hausfather (2020). This series is available at `https://berkeley-earth-temperature.s3.us-west-1.amazonaws.com/Global/Land_and_Ocean_summary.txt`.
- The Goddard Institute for Space Studies (GISS) Surface Temperature Analysis (GISTEMP) at the National Aeronautic Space Administration (NASA) (Lenssen et al., 2019). This series is available at `https://data.giss.nasa.gov/gistemp/tabledata_v4/GLB.Ts+dSST.txt`.
- Dynamically Consistent ENsemble of Temperature Interpolated (DCENT-I) (Chan et al., 2026). This series is available at `https://dcent-i.github.io/`.

Anomalies are computed relative to the period 1850-1900.

1.2 Data and parameter uncertainties

1.2.1 Observational uncertainty

To quantify uncertainty in our results arising from the underlying data, we downloaded and analyzed all members of the DCENT-I and HadCRUT observational ensembles (these are not available for the other datasets). More specifically, the two ensembles included here are:

- The dynamically Consistent ENsemble of Temperature Interpolated (DCENT-I) (Chan et al., 2026). The gridded individual members were downloaded from `https://dcent-i.github.io/`.
- The Hadley Centre/Climatic Research Unit, Version 5 (HadCRUT), gridded surface temperature (Morice et al., 2021). Individual ensemble members are available at `https://www.metoffice.gov.uk/hadobs/hadcrut5/data/HadCRUT.5.1.0.0/download.html`.

Analyzing each ensemble member allows us to propagate data uncertainty through the changepoint detection procedure. Figure S2 presents uncertainties in regional changes in trend in the DCENT-I ensemble. The agreement across ensemble members tend to be larger in most regions selected in the main paper such as Southeast Mexico, Gulf of Mexico, East Mediterranean, Southeast China, New Zealand and North Pacific, except for Bolivia where agreement across ensemble members is approximately 50% (Figure S2A). The median timing of changepoints is consistent with findings in the main paper, and uncertainty in changepoint timing is rather small in the seven regions identified and becomes large at high latitudes where observations are more sparse (Figure S2B-C).

Figure S3 presents uncertainties in regional trend changes in the HadCRUT ensemble. Agreement across ensemble members is strong in the North Pacific, Bolivia, East Mediterranean and New Zealand, whereas Southeast Mexico, Gulf of Mexico and Southeast China have less than 50% agreement across ensemble members (Figure S3A). The median timing of changepoints is consistent with regions identified in the main paper, but the number of grid cells with changes is larger across the hadCRUT ensemble members (Figure S3B). Uncertainty in changepoint timing tends to be smaller in the seven regions identified and is the largest at high latitudes in the southern hemisphere (Figure S3C).

1.2.2 Parameter uncertainty

In the main, we provide confidence sets on regional averages based on the Berkeley dataset (Figure 4). We include here the confidence sets calculated at a grid cell level, only for the grid cells where one changepoint was detected (Figure S4). The length of confidence intervals is on par with regional averages presented in Figure 4.

1.3 Sensitivity analysis

1.3.1 Reduced penalty

In the main, we analyzed the gridded temperature datasets with a BIC penalty:

$$P(m) = (4m + 4)\ln(N), \tag{1}$$

where m represents the number of changepoints and N is the length of the time series. This penalty explicitly accounts for the changepoint timing alongside the different segment's parameters (the intercept, slope, and autocorrelation coefficient). Here, we also include an analysis on the Berkeley dataset with a reduced penalty that does not count the changepoint timing in the penalty term:

$$P(m) = (3m + 4)\ln(N). \tag{2}$$

This penalty is less conservative than the BIC penalty used in the main paper 1 and consequently leads to more changes detected. We use this reduced penalty instead of an Akaike Information Criterion (AIC) penalty because the AIC is inconsistent in changepoint settings Zheng et al. (2022). The regional changes identified in the Berkeley surface temperature dataset are presented in Figure S5. With a reduced penalty, the main regions identified in Figures 1-2 in the Main expand, and new regions appear that were negligible with a higher penalty. More specifically, the number of grid cells with at least one detected change increased from 5.9% to 19% for the Berkeley dataset.

1.3.2 False positive rates

To assess the false positive rates of our changepoint detection technique, we generated 1000 random synthetic series of 55 data points (covering 1970-2024) from a first-order autocorrelation (AR(1)) process

$$\epsilon_t = \phi\epsilon_{t-1} + Z_t, \tag{3}$$

where $\{Z_t\}$ is independent and identically distributed Gaussian noise with a mean of 0 and variance $\sigma^2 = 0.01$ and $\phi = 0.23$. A long-term trend of 0.02 ^oC per year was superimposed. Those values were obtained from the Berkeley global mean surface temperature time series. We analyzed the synthetic time series using a BIC penalty as used in the main (Equation 1) and a reduced penalty included above (Equation 2). Table S1 presents the rate of false detections obtained with both penalties. With the BIC penalty, the false positive rate is smaller, as expected.

1.3.3 True positive rates

To assess the detection power of the changepoint technique, we generated random synthetic series of 55 data points from a first-order autocorrelation (AR(1)) process as in Equation

3 with with a mean of 0 and variance $\sigma^2 = 0.01$ and $\phi = 0.23$, with a superimposed long-term trend of 0.02 °C per year. Then, we impose changepoints at times 10, 20, 30 and 40, corresponding to the start of a new regime at years 1980, 1990, 2000 and 2010, representing the different timings detected on gridded surface temperature data. The magnitude of the changepoints is set to vary to represent the range of changepoint energy (E) observed on the surface temperature datasets (Table 2 in the Main) with E varying from 0.25 (small E, difficult to detect) to 3 (large E, easy to detect). We analyze the time series generated with our changepoint model and compute the proportion of time series for which a change in trend is detected when a change is present. The results are presented in Figure S6. For a fixed E, the power is slightly higher when the changepoint is closer to the middle of the time series and decreases if the changepoint moves towards the beginning or end of the time series. As such, changes that took place in the 1990s and 2000s are easier to detect than in the 2010s or 1980s. The detection power of the technique utilized here increases rapidly with the changepoint energy: when $E = 0.5$ changes will likely be detected (probability > 0.75) except if they occur in 1980, then there is a reduced detection power (Figure S6A). With the reduced penalty, the power of detection increases as expected. When $E = 0.5$, changes will most likely be detected (probability > 0.9) except if they occurred in 1980 when the power is reduced (Figure S6B). Overall, this power analysis demonstrates the skill of the method used here in detecting a change in trend when there is one.

1.3.4 Quadratic trend

An alternative representation for an acceleration in warming is a quadratic trend model Richardson (2022). Here we also analyze a gridded surface temperature dataset with a quadratic regression model fitted over the period 1970-2024:

$$X_t = \alpha_1 + \beta_1 t + \beta_2 t^2 + \epsilon_t, \tag{4}$$

where α_1 is the intercept, β_1 and β_2 represent the linear and quadratic trend components and ϵ_t is the errors that we model with a first-order autocorrelation model.

Figure S7 presents the estimates and significance of quadratic trends. Regions identified by the changepoint detection method applied to the Berkeley dataset in Figures 1A and 2A are also flagged here. However, with the quadratic trend there are more grid cells with a detected acceleration than with the piecewise model with a BIC penalty used in the Main. We note that these two models represent different definitions of an acceleration (smooth vs piecewise linear), and different results are to be expected.

Supplementary Tables and Figures

Tables

Table S1: False positive rates (%) obtained by applying the changepoint technique to 1000 random time series with two different penalties.

Penalty	Rate of false detection (%)
(4m+4)ln(N)	1.8
(3m+4)ln(N)	9.4

Figures

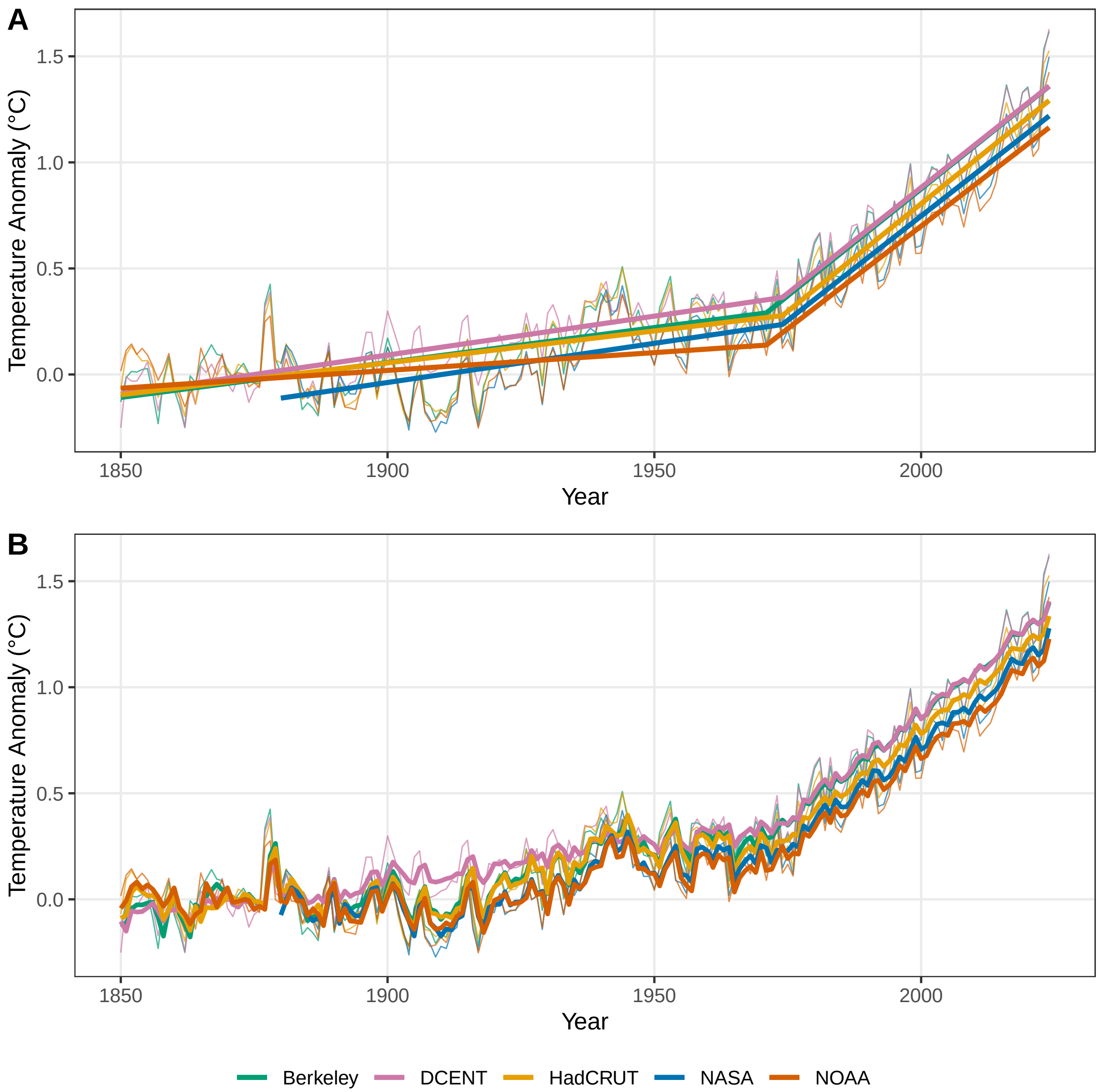

Figure S1: The NASA, HadCRUT, NOAA, Berkeley and DCENT-I series from 1850-2024 averaged globally. Piecewise linear regression fits are superimposed and showing A) Trend only and B) Trend and autocorrelation.

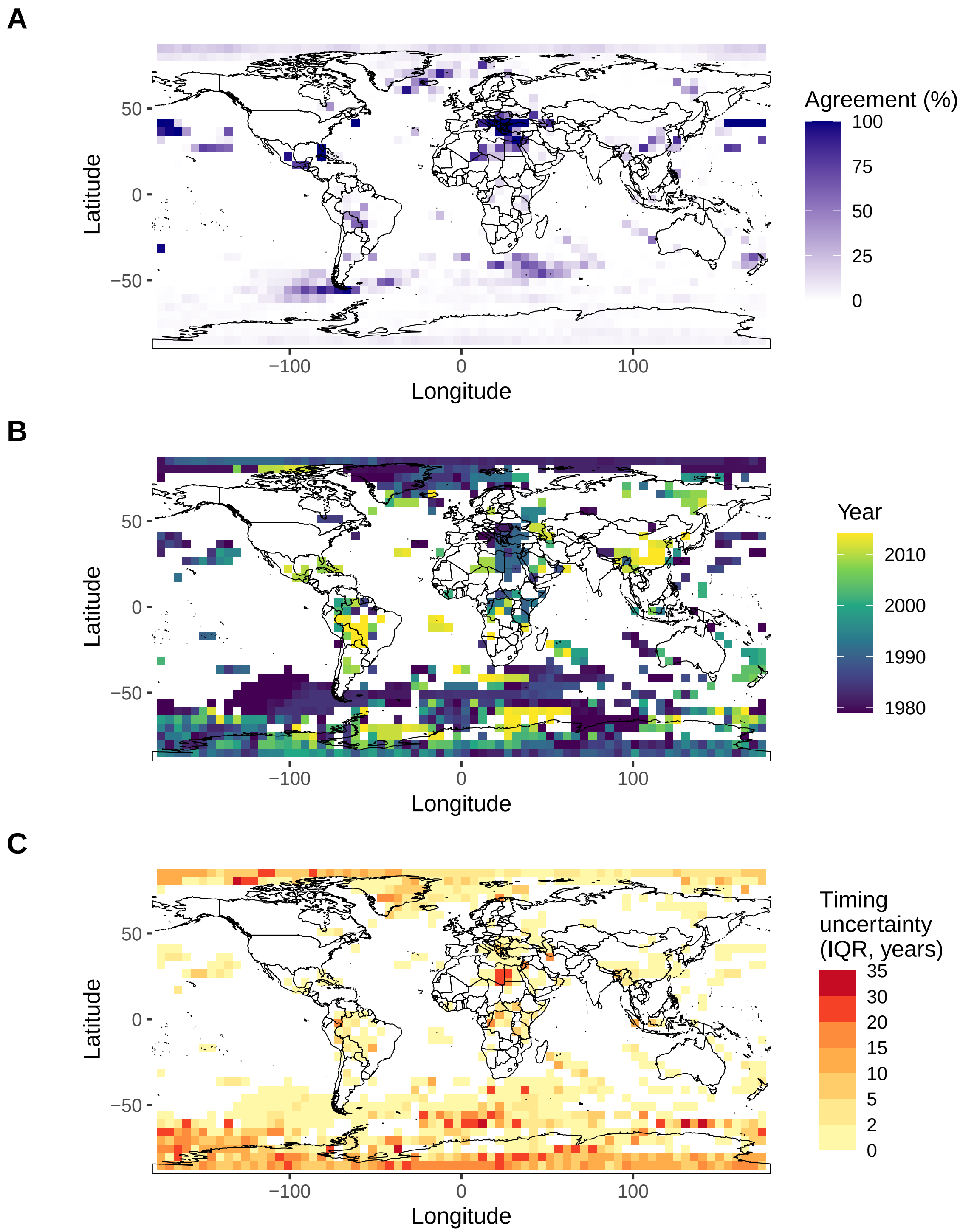

Figure S2: Changepoint analysis of the DCENT-I ensemble. A) Agreement amongst the 200 ensembles members about the presence of a changepoint, B) Median year of changepoint detected across ensemble members and C) Interquartile range (IQR) of the changepoint timing across all ensemble members.

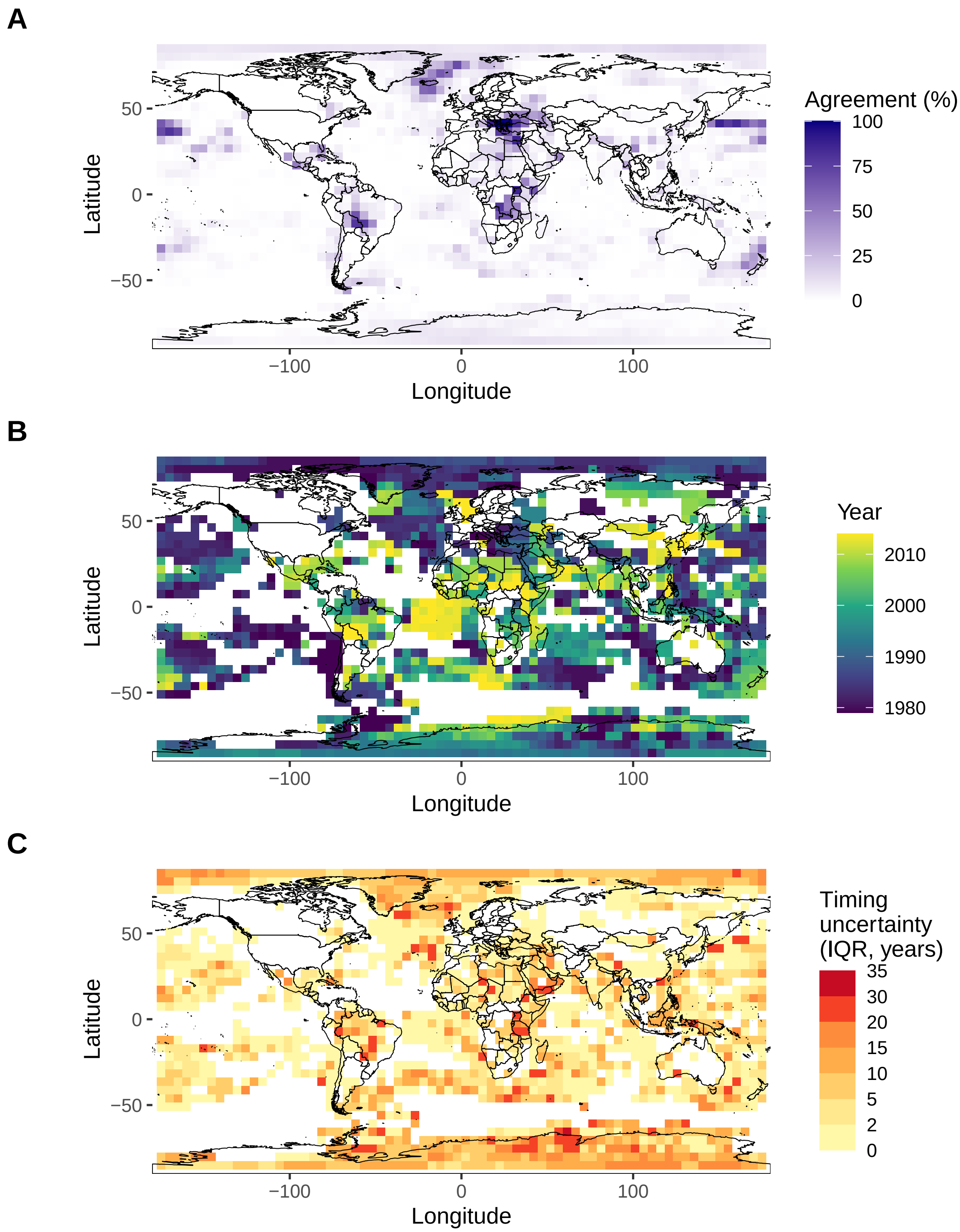

Figure S3: Changepoint analysis of the HadCRUT ensemble. A) Agreement amongst the 200 ensembles members about the presence of a changepoint, B) Median year of changepoint detected across ensemble members and C) Interquartile range (IQR) of the changepoint timing across all ensemble members.

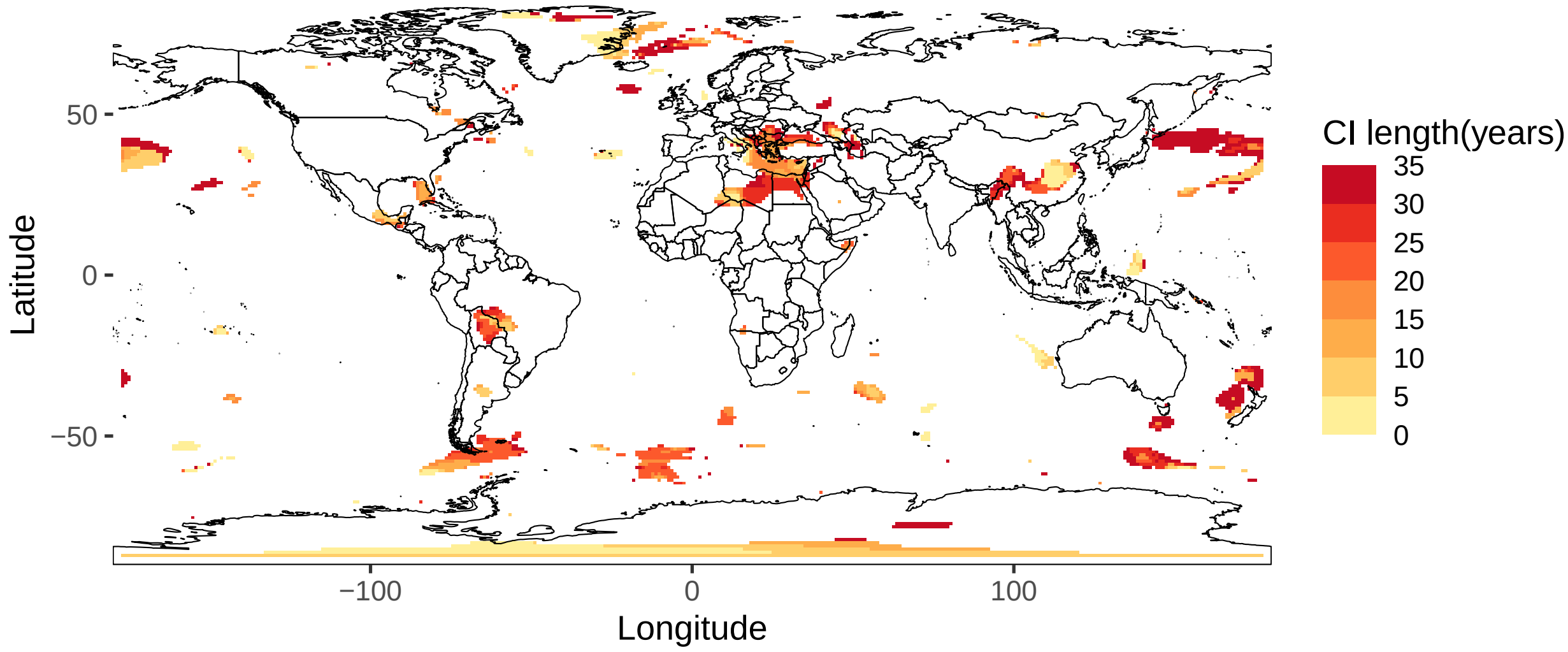

Figure S4: Length of confidence intervals for changepoint timings for grid cells where one changepoint was detected, using the Berkeley dataset.

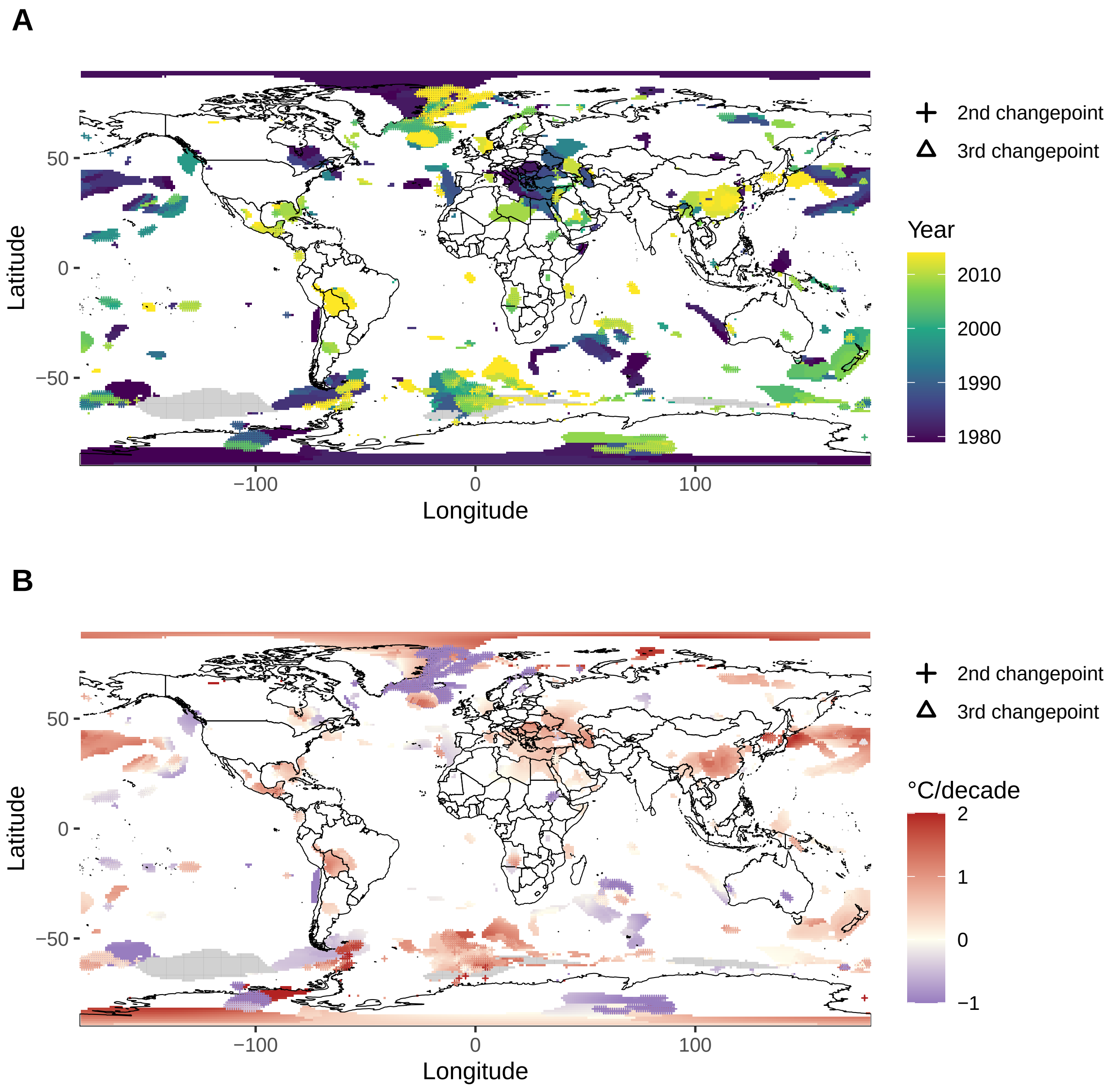

Figure S5: Regional changes in the rate of warming since 1970 detected with a reduced penalty and using the Berkeley dataset. A) Timings of changes detected and B) Magnitude of changes detected. White areas indicate where changes are not detectable and grey areas indicate missing data. When more than one changepoint is detected, they are labelled with crosses and triangles.

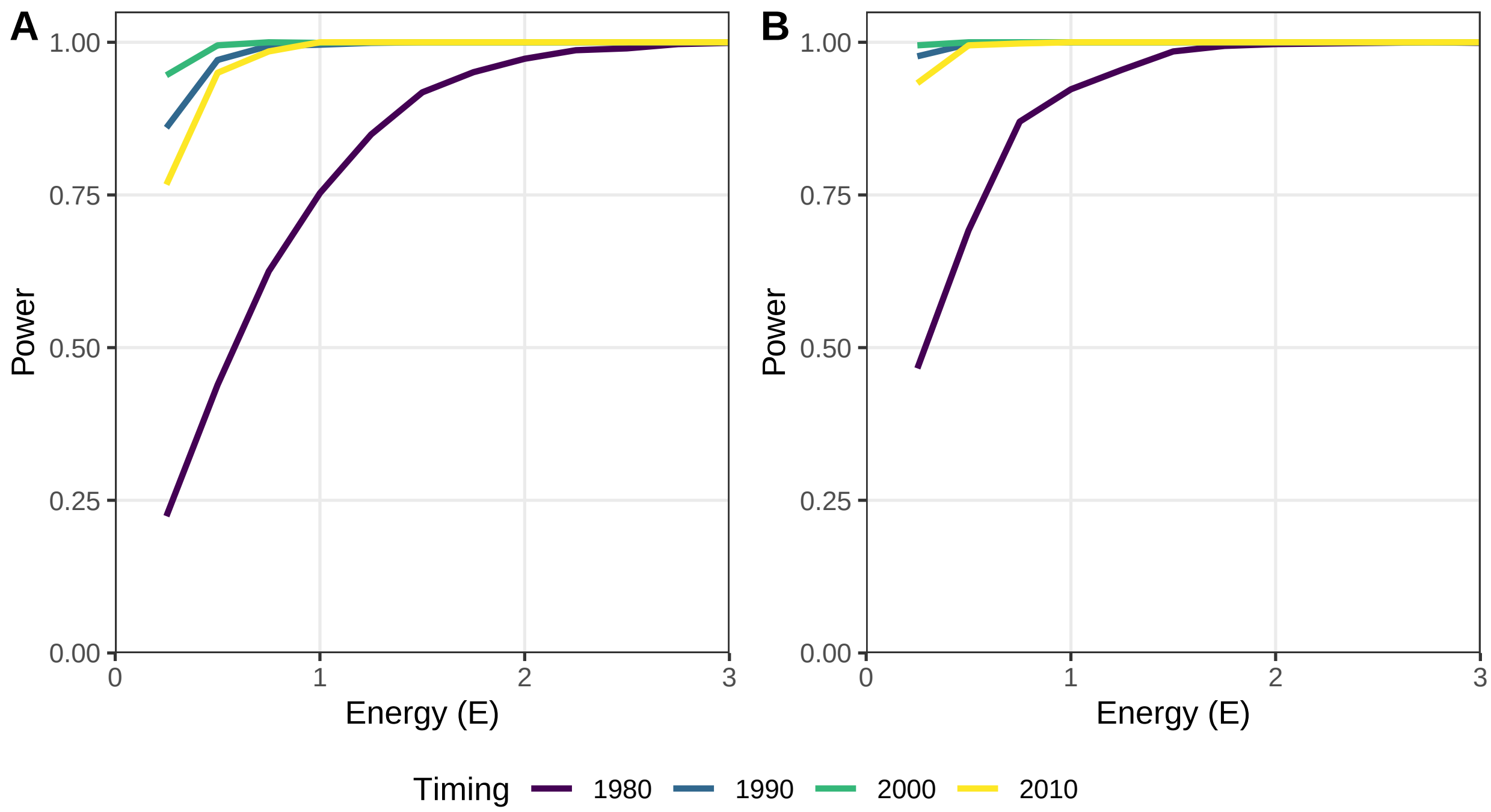

Figure S6: True positive rates of the changepoint detection technique depending on changepoint energy (E) A) with a BIC penalty Equation (4m+4)ln(N) and B) with a reduced penalty (3m+4)ln(N). Note that the false positives are presented in Table S1.

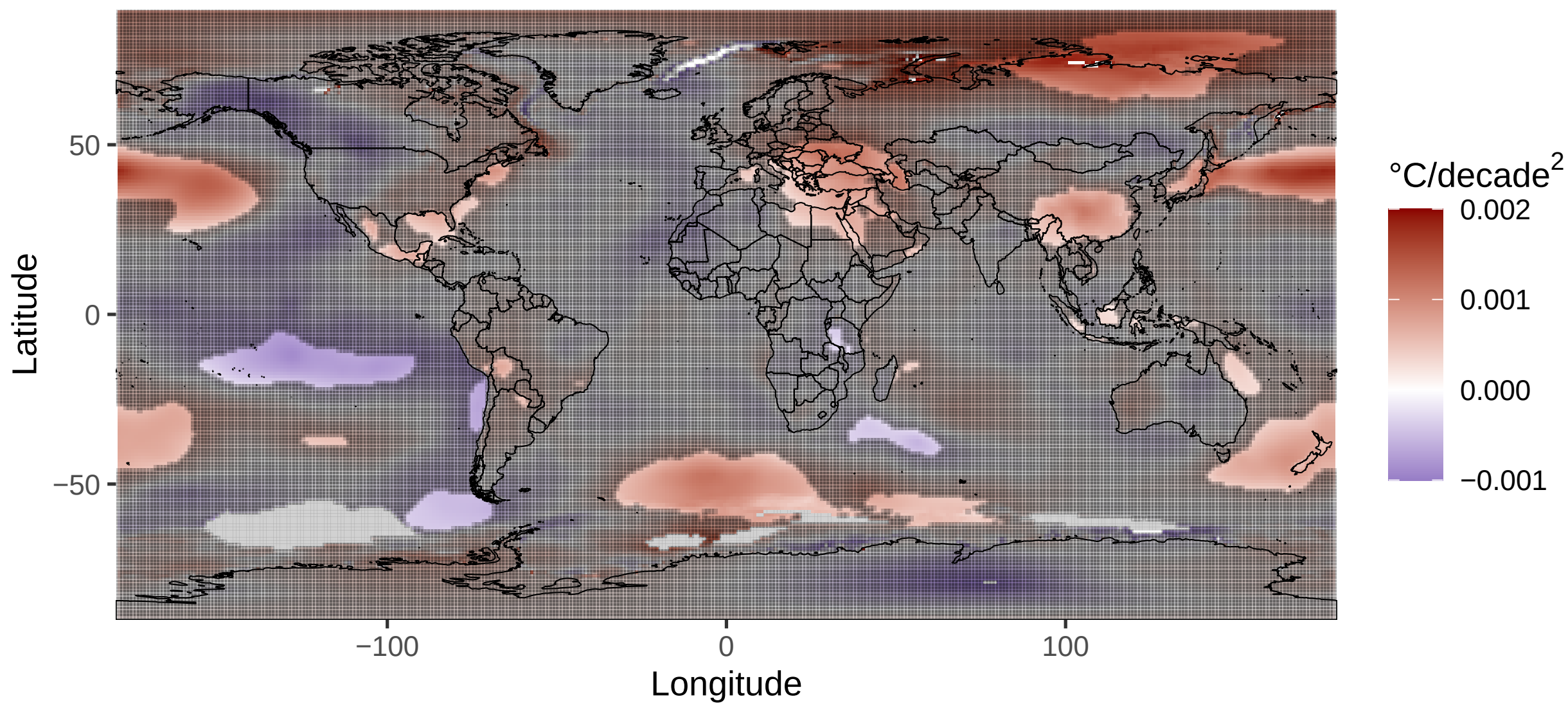

Figure S7: Smooth changes in the rate of warming since 1970 represented by a quadratic trend (Berkeley dataset). Stipling indicates where the quadratic trend component is not significantly different from zero (p-value > 0.05). Grey indicates missing data.

Supplementary References

Beaulieu, C., Gallagher, C., Killick, R., Lund, R., and Shi, X. (2024). A recent surge in global warming is not detectable yet. *Communications Earth & Environment*, 5(1):576.

Chan, D., Chan, S. C., Siddons, J. T., Cable, A., Faulkner, A., Cornes, R. C., Kent, E. C., Gebbie, G., and Huybers, P. (2026). DCENT-I: A globally infilled extension of the dynamically consistent ENsemble of temperature dataset. *Geoscience Data Journal*, 13(2):e70054.

Lenssen, N. J. L., Schmidt, G. A., Hansen, J. E., Menne, M. J., Persin, A., Ruedy, R., and Zyss, D. (2019). Improvements in the GISTEMP uncertainty model. *Journal of Geophysical Research: Atmospheres*, 124(12):6307–6326.

Morice, C. P., Kennedy, J. J., Rayner, N. A., Winn, J. P., Hogan, E., Killick, R. E., Dunn, R. J. H., Osborn, T. J., Jones, P. D., and Simpson, I. R. (2021). An updated assessment of near-surface temperature change from 1850: The HadCRUT5 data set. *Journal of Geophysical Research: Atmospheres*, 126(3):e2019JD032361.

Richardson, M. T. (2022). Prospects for detecting accelerated global warming. *Geophysical Research Letters*, 49(2):e2021GL095782.

Rohde, R. A. and Hausfather, Z. (2020). The Berkeley Earth land/ocean temperature record. *Earth System Science Data*, 12(4):3469–3479.

Vose, R. S., Huang, B., Yin, X., Arndt, D., Easterling, D. R., Lawrimore, J. H., Menne, M. J., Sanchez-Lugo, A., and Zhang, H. M. (2021). Implementing full spatial coverage in NOAA's global temperature analysis. *Geophysical Research Letters*, 48(4):e2020GL090873.

Zheng, C., Eckley, I., and Fearnhead, P. (2022). Consistency of a range of penalised cost approaches for detecting multiple changepoints. *Electronic Journal of Statistics*, 16:4497–4546.
