# Supplementary material for: Space-time signatures of surface warming accelerations since 1970
Source: Nat Commun. 2026 Sep 22;17:9742. doi: 10.1038/s41467-026-77514-z (PMC13598101; doi:10.1038/s41467-026-77514-z)
Supplement: Supplementary file 2 [file 41467_2026_77514_MOESM2_ESM.pdf]

nature portfolio

Peer Review File

Space-time signatures of surface warming accelerations since 1970

Corresponding Author: Professor Claudie Beaulieu

This file contains all reviewer reports in order by version, followed by all author rebuttals in order by version.

Version 0:

Reviewer comments:

Reviewer #1

(Remarks to the Author)
By presenting a statistical analysis of which areas of the Earth show accelerated warming in observations, this work would be a meaningful addition to discussions about possible "accelerations" in global warming. The authors show that some regions of the Earth show detectable accelerations in warming since 1970, especially in the northern hemisphere mid-latitudes. These areas, however, are a relatively small part of the overall area of the Earth, so it seems probable that regionally specific changes in aerosol forcing, rather than greenhouse gas increases, may have driven some of these regional accelerations. The manuscript is well organized, well written, and easy to comprehend.

I have outlined some suggestions below. Mainly, I think that including polar regions and some discussion of the land/ocean breakdown of warming accelerations would improve this work's relevance and utility to the wider community. I have also included some suggestions to clarify and contextualize the results already presented, as well as line-by-line comments.

Polar regions: Polar amplification is one of the main ways in which we expect the rate of warming to vary spatially across the globe, and you mention some of its drivers in the introduction. I think it would be important to include polar regions in this analysis. If the global temperature datasets use different definitions of the "surface" for sea ice areas (do they, or is this only true for SST datasets?), you could exclude polar regions from the aggregated dataset but show global results for the individual datasets which include global surface temperatures.

Land vs. Ocean: What percentage of land vs. ocean grid cells show a detectable acceleration in warming? This would be a meaningful and interesting piece of analysis to include: how much do we see "land amplification" reflected in these results? It would be helpful to see Figure 3 split into land/ocean parts.
Data uncertainty: Some more discussion of the uncertainties in the underlying datasets would be helpful. What are the differences in regional trends between the different global temperature datasets? Do the datasets themselves report uncertainty ranges for grid-point temperature estimates? It may be worth noting that Menemenlis et al. (2025) find that there are large differences in spatial-average trends across SST datasets, but a much smaller difference in average trends across the global (combined land+ocean) datasets that you use here, because these global datasets use relatively similar SSTs.

Internal variability: As you mention, processes in the Earth system give rise to long timescale memory, and known modes of variability such as the PDO have identifiable spatial patterns. The discussion section focuses on aerosols, and cites some examples of possible links between aerosol reductions and sections where change-points are detected. However, is there also a potential contribution to these patterns from modes of internal variability? While fully exploring this question is outside the scope of the paper, it warrants a more robust discussion.

Quadratic trends: Figure S4 is a nice complement to the analysis in the main text. It corroborates many of the areas where piecewise warming accelerations are detected, and shows regional coherence. It's also interesting because it includes a significant decelerating trend in the cooling eastern Pacific, which is not detected in the change-point analysis. Why?

Clarification: In general, when you write about "accelerations in warming," does this include any detectable increases in the rate of change of temperature, including from cooling to warming? My understanding is that some areas or grid cells, for example the North Pacific area as shown in Figure 4H, may have detectable negative trends during some part of the time period.

Line-by-line comments:
11-12: Slight clarification needed, as Rantanen & Laaksonen focused on the specific single event of the September 2023 warming “spike”, and this sentence seems to refer to warming “spike” events in general.
13-15: Here it would be worth noting the reduction in shipping tracks due to the 2020 International Maritime Organization regulation that limited sulfur in shipping fuel (e.g. Gettelman et al., 2024), as this would be specifically relevant to the 2023-2024 “spike”.
29-30: It’s a common misconception that the primary reason for land/ocean warming contrast is due to variable heat capacities. While this is a factor, it is not the main factor, and the land-ocean contrast appears in the equilibrium response to warming (Manabe et al., 1991; Sutton et al., 2007).
31: As you mention high latitude processes here, it would be nice to include some analysis of high latitudes in this paper, even if it’s only with individual datasets rather than an aggregate.
42: Why did you choose to start in 1970?
59: Instead of “experienced a change” do you mean “experienced a detectable change”?
75-76: Is there a previous understanding / reference for why the Southern Ocean saw decelerations in the 1980s?
80: Was there a particular threshold for the spatial extent, or for the level of consistency across datasets, of regions with detectable accelerated warming that you used to select these areas? And, can you clarify whether the timeseries shown in Figure 4 include the entire boxed area, or just the grid cells with detectable warming accelerations?
128: Interesting that Figure S4 includes significant negative quadratic trends in the eastern tropical Pacific, but no such decelerations are detected during the changepoint analysis. While other areas—including the major warming accelerating areas—show more similarities between the two methods. Why do you think this is? Would it be worth pointing this out?
148-151: It doesn’t seem necessary to completely exclude polar regions from this analysis: see comment above.
238: A major change in shipping regulations came in 2020, so would be relevant to warming trend since that year.
247-249: Is it possible that some of the regional accelerations could have a significant contribution from internal climate variability, not just external aerosol forcing? Though it’s outside the scope of this study, one way to test this would be to perform an analysis similar to what you did in this manuscript on unforced climate model output.
Figure 2: I understand “magnitude of regional changes in the rate of warming” to mean, for areas where piecewise changes are detected, the difference between the slopes of each piece.
Figure 4: It’s interesting that Area H shows a change from cooling to warming trend—when I read “acceleration in warming” I assumed that these areas would all change from slow warming to faster warming, not cooling to warming. Would be worth clarifying.
Figure S4 caption: Clarify which dataset used here? Also Berkeley?

References:
Gettelman, A., M. W. Christensen, M. S. Diamond, et al. “Has Reducing Ship Emissions Brought Forward Global Warming?” Geophysical Research Letters 51, no. 15 (2024): e2024GL109077. https://doi.org/10.1029/2024GL109077.
Manabe, S., R. J. Stouffer, M. J. Spelman, and K. Bryan. “Transient Responses of a Coupled Ocean–Atmosphere Model to Gradual Changes of Atmospheric CO2. Part I. Annual Mean Response.” Journal of Climate. Journal of Climate 4, no. 8 (1991): 785–818. https://doi.org/10.1175/1520-0442(1991)004%253C0785:TROACO%253E2.0.CO;2.
Menemenlis, Sofia, Gabriel Vecchi, Wenchang Yang, Stephan Fueglistaler, and Shiv Priyam Raghuraman. “Consequential Differences in Satellite-Era Sea Surface Temperature Trends Across Datasets.” Preprint, In Review, February 4, 2025. https://doi.org/10.21203/rs.3.rs-5493841/v1.
Sutton, Rowan T., Buwen Dong, and Jonathan M. Gregory. “Land/Sea Warming Ratio in Response to Climate Change: IPCC AR4 Model Results and Comparison with Observations.” Geophysical Research Letters 34, no. 2 (2007). https://doi.org/10.1029/2006GL028164.

(Remarks on code availability)

Reviewer #2

(Remarks to the Author)
Space-time signatures of surface warming accelerations since 1970

Beaulieu et al.
Nature Communications manuscript
Review by Manfred Mudelsee (29 October 2025)

Overall assessment

This is a good and interesting manuscript, which would fit well into Nature Communications. The subject—rates of change of surface temperatures since 1970 across five gridded datasets—is of high scientific interest, particularly given the ongoing discussion about “global warming acceleration.” The method (piecewise linear regression) is sound, and the presentation (text, figures, mathematical details) is generally adequate.

I recommend publication after three minor revisions are addressed. I first detail those three points, followed by several additional comments where a brief author response or small improvement would be beneficial.

Thank you for letting me review this work—I would be pleased to see the revised version.

Minor revisions

(A) Uncertainty quantification for change-point parameters
You perform piecewise linear regressions with an arbitrary number (m) of change-points, selecting m via the BIC criterion. This is essentially a statistical test for the number of linear segments, yielding slope estimates. The assumed data-generating process (piecewise linear plus AR(1) noise) is appropriate for temperature time series.

However, the analysis would be strengthened by including uncertainty estimates (standard errors or confidence intervals) for the change-point times and slopes. I previously developed a related "break regression" method for m = 1 (Mudelsee 2009) using weighted least squares and moving-block bootstrap resampling of residuals. In my book (Mudelsee 2014, § 4.2.2), I examined bootstrap BCa confidence intervals and their coverage via Monte Carlo experiments.
I recommend that you consider providing uncertainty ranges for your estimated change-point parameters—either by using the BREAKFIT tool (Mudelsee 2009, 2014) or by extending co-author Lanzante's software.

(B) Fairness toward prior work / citation completeness
To acknowledge previous developments and broaden the readership's context, please cite the following two works:

Mudelsee M (2009) Break function regression: A tool for quantifying trend changes in climate time series. Eur Phys J Spec Top 174: 49–63.

Mudelsee M (2014) Climate Time Series Analysis: Classical Statistical and Bootstrap Methods, 2nd ed. Springer, Cham, xxxii + 454 pp.

(C) Spatial dependence
This is admittedly a difficult issue. Nature exhibits memory not only in time (your AR(1) noise model) but also in space. Yet this aspect seems neglected (see line 157). Intuitively, ignoring spatial dependence may reduce the power of tests for post-1970 warming accelerations, but by how much? A Monte Carlo experiment could provide insight.
At minimum, please discuss the implications of spatial dependence, and if feasible, include a quantitative analysis.

Additional comments

Line 51: Provide a reference for long-memory models and clarify that their autocorrelation function decays hyperbolically, not exponentially as in short-memory (AR(1)) processes.

Line 101: Write "SNR = 2.6" (with spaces).

Line 109: The statement "BIC penalty has been proven to perform best …" seems overly general. While you cite a reference, "best" depends on analytical goals (e.g., power, robustness to distributional shape or noise model). Please note such caveats.

Lines 153–157: Clarify that you work with evenly spaced time series. (Uneven spacing is common in paleoclimate studies; see Mudelsee 2014 for discussion.)

Line 169: Allowing error-process parameters to change at the change-points is interesting but perhaps not parsimonious. Consider or at least discuss a stationary-error alternative.

Line 179 ("Brute force"): For m = 1, the break method (Mudelsee 2009) remains computationally feasible even with bootstrap resampling. The change-point time can be restricted to observed time values, provided its uncertainty is similar to or larger than the temporal spacing near the break. The claim that PELT "guarantees the optimal solution" seems overstated unless the entire parameter hyperspace is explored—please clarify.

Eq. (5), lines 191–192: Dimensional consistency is unclear. Parameters β_2 (or β_1) have units °C / decade, whereas σ_2 has units °C, making their ratio (the defined SNR) dimensional. Yet you present SNR as dimensionless (cf. line 101). Please revise or explain. In general, standard errors or CIs may convey estimation accuracy more transparently than SNR.

References: Require careful reformatting (e.g., "ENSO," capitalization in #6, incomplete author list in #4 – who is "A.P."?).

Figure 1: Distinguish "no data" (white) from "not detectable" (grey) instead of using one grey shade for both.

Figure 3 caption: The phrase "x-axis" actually refers to the double horizontal axis showing percentages of detected change-points—please clarify.

Figure 4: Panels B–H show m = 1. How many grid cells have m = 1, 2, or higher? A visual summary would be informative. Given your post-1970 focus, m = 1 likely dominates, in which case the break model (Mudelsee 2009) would be an excellent benchmark.

Supplementary Information line 56: "°C" should not be italicized (easily fixed in LaTeX).

Supplementary Fig. S4: The quadratic-term results are interesting—perhaps emphasize them more in the main text.

(Remarks on code availability)

Reviewer #3

(Remarks to the Author)
This is a well-posed paper that uses piecewise fits to gridded surface temperature datasets to identify significant change points that can be interpreted as accelerations or deccelerations in the temperature time series at each grid point. The results demonstrate some regional accelerations, mostly in the midlatitudes of the Northern Hemisphere, and a mix of accelerations and deccelerations in the midlatitudes of the Southern Hemisphere. The topic is important and timely as there has been discussion of possible warming acceleration, and this paper provides a rigorous assessment of those regions in which this may be evident.

The analysis is sound and the authors have been clear in description of their methods and in highlighting limitations of the methods and checking, where possible, on the limitations. For example, the authors use multiple different gridded datasets to assess observational uncertainty. The authors are clear that their modelling of the surface temperature data does not account for long memory processes, and correctly note that this is more a limitation/caution over the oceans than over land areas. While they identify this as an issue for the North Pacific Ocean, this would also apply to the midlatitude acceleration/decceleration regions in the Southern Hemisphere, which are mostly over the Southern Ocean. The authors have not really remarked on the Southern Hemisphere results here, which are in partial contrast to those in the Northern Hemisphere.

The paper is well written and provides sufficient detail to be reproduced. I can not identify any issues with the interpretation of the results, and their conclusions are all consistent with what they have shown. While that might seem unremarkable, it is refreshing to see. This paper surpasses the standards in the field, and is in my view, appropriate for publication, and makes a worthwhile contribution to current understanding of the expression of climate change.

(Remarks on code availability)

Version 1:

Reviewer comments:

Reviewer #1

(Remarks to the Author)
The authors have addressed my concerns from the previous review. The additional clarifications and useful improvements to the discussion bring the manuscript into great shape for publication.

I have one remaining suggestion, which is to clarify the confusing wording on lines 16-20. There, the effect of ship track aerosol reductions is mentioned at the end of the 2nd paragraph of the introduction. Then, the 3rd paragraph begins, "another explanation for the exceptional temperatures..." and again invokes the effect of ship track aerosol reductions. The framing here is redundant and confusing.

Provided this small problem is corrected, I believe the manuscript is ready for publication.

(Remarks on code availability)
The code appears well-documented and well-organized. However, I did not download the datasets or run the code myself.

Reviewer #2

(Remarks to the Author)
My main concern remains unresolved.

The manuscript still does not provide uncertainty estimates (e.g. confidence intervals or standard errors) for the estimated change-point times and slope parameters. This is a central issue, as the main conclusions about regional warming accelerations depend directly on the reliability of these estimates. Without such uncertainty quantification, the statistical strength of the results cannot be properly assessed.

I do not find the authors’ argument convincing that uncertainty in the number of change-points would dominate and therefore make parameter uncertainty less relevant. At least for the frequent case of $m = 1$, uncertainty estimation for change-point timing and slopes would be straightforward and feasible with existing methods.

As an aside, I note that the distinction made between “model selection” and “statistical testing” does not remove the

inferential nature of the procedure. The manuscript itself refers to detection rates and power, and the selected number of change-points is clearly subject to uncertainty given finite and noisy data.

For these reasons, I do not consider the manuscript sufficiently substantiated in its present form.

(Remarks on code availability)

Version 2:

Reviewer comments:

Reviewer #2

(Remarks to the Author)
The revised manuscript has improved, and I appreciate that the authors have now added confidence intervals for changepoint timings in the selected regional averages with one changepoint. This is a useful addition.

However, my central concern is only partially addressed. The manuscript still does not provide uncertainty estimates for the changepoint parameters at the grid-cell level, although Figures 1 and 2 are built around grid-cell changepoint timings and slope changes. In particular, no uncertainty estimates are provided for the slope parameters or for the slope changes, although these are central to the interpretation of warming acceleration.

The newly added confidence intervals are conditional on a single-changepoint model and are provided only for regional averages. This is helpful, but it does not fully address the uncertainty of the main gridded results. Moreover, the method used for these confidence intervals relies on a submitted manuscript (co-authered by Rebecca Killick, a co-author of the present manuscript), and the present paper gives only a brief description of the procedure. I therefore find it difficult to assess how reliable these intervals are, including their coverage properties.

The additional analyses based on observational ensembles are also useful, but they address data uncertainty rather than the parameter uncertainty of the changepoint model itself. This was not the main point of my previous concern.

Overall, I regard the manuscript as improved and scientifically interesting. However, I still do not think that the uncertainty of the main changepoint results is quantified at the level needed for a fully convincing statistical assessment. I leave it to the editor to decide whether this remaining limitation is acceptable for publication in Nature Communications.

(Remarks on code availability)

Reviewer #4

(Remarks to the Author)
The revised manuscript presents an interesting and sound analysis. The question whether global warming is accelerating is definitively of interest.

The authors make several assumptions, e.g., the piecewise linear model, AR[1] residuals, BIC penalty. All these assumptions are made transparent, are discussed and the choices seem well justified.

Choice of the penalty

The authors admit that the results are highly dependent on the choice of their penalty (BIC), which is definitively a weak point, which has been made transparent. However, I cannot follow their sensitivity study. I do not see the point in changing the penalty from (4m+4) to (3m+4). My first idea in this respect would be to use the general penalized likelihood setup with a regularization parameter \lambda and use a few different \lambdas in a sensitivity study. Maybe the authors can motivate their choice here.

The quadratic model

The authors commented on their alternative model using a linear plus quadratic model that it does not provide a specific timing. While this is true, an accelerating trend does not necessary need a specific timing unless the model is bound to two distinct linear trends. The quadratic model shows acceleration w/o specific timing. I'd suggest to change the wording

Autocorrelated errors

It might be worth mentioning that the likelihood function does not easily factorize for autocorrelated errors. I suggest explaining this at least in the supplementary information.

Signal to noise ratio

I would have chosen another name for the concept shown as it gives a weighted difference of two SNRs.

Regional averages

It is well explained how the average timing has been obtained when aggregating different data sets. But how is this done for the regions given in Fig. 4? Maybe I missed that. Is it the same grid-point wise analysis and then averaging of the timing? How are then CIs treated? Or are the time series averaged over the regions first? (Than the CI problem is clearer).

Confidence intervals (CI)

Confidence intervals are given conditioned on the choice of 1 break point. It might be noteworthy to explain why this is the case (penalized likelihood). Furthermore, the calculation of CIs has been deferred to an unpublished paper (60). I would expect that at least the idea should be formulated here. Furthermore, only the CIs of the timing are given (and only exemlarily for some interesting regional averages), not the CIs for the slopes as it has been asked by another reviewer.

Smaller comments
- how is the regridding done
- l. 203 "... work have shown..."
- the caption of Fig. 4 mentions a panel l which does not exist

(Remarks on code availability)

Open Access This Peer Review File is licensed under a Creative Commons Attribution 4.0 International License, which permits use, sharing, adaptation, distribution and reproduction in any medium or format, as long as you give appropriate credit to the original author(s) and the source, provide a link to the Creative Commons license, and indicate if changes were made.
In cases where reviewers are anonymous, credit should be given to 'Anonymous Referee' and the source.
The images or other third party material in this Peer Review File are included in the article's Creative Commons license, unless indicated otherwise in a credit line to the material. If material is not included in the article's Creative Commons license and your intended use is not permitted by statutory regulation or exceeds the permitted use, you will need to obtain permission directly from the copyright holder.
To view a copy of this license, visit https://creativecommons.org/licenses/by/4.0/

Response to Reviewers

"Space-time signatures of surface warming accelerations since 1970"
by Beaulieu, Johnson, Killick, Lanzante, and Knutson
Nature Communications

We thank the three reviewers for taking time to carefully assess our manuscript and for providing constructive comments, which have substantially improved the manuscript. We have carefully revised the manuscript to address the reviewers' comments and prepared point-by-point responses. Below, we present the detailed responses to the reviewers' comments. Referees comments are in plain text and our responses in italic.

Reviewer 1

Main Comments:

By presenting a statistical analysis of which areas of the Earth show accelerated warming in observations, this work would be a meaningful addition to discussions about possible "accelerations" in global warming. The authors show that some regions of the Earth show detectable accelerations in warming since 1970, especially in the northern hemisphere mid-latitudes. These areas, however, are a relatively small part of the overall area of the Earth, so it seems probable that regionally specific changes in aerosol forcing, rather than greenhouse gas increases, may have driven some of these regional accelerations. The manuscript is well organized, well written, and easy to comprehend.

I have outlined some suggestions below. Mainly, I think that including polar regions and some discussion of the land/ocean breakdown of warming accelerations would improve this work's relevance and utility to the wider community. I have also included some suggestions to clarify and contextualize the results already presented, as well as line-by-line comments.

Response: *We thank the reviewer for their positive opinion on our manuscript. We modified the manuscript to include polar regions in the analysis and to break down the changes detected over land vs ocean. We provide more details in our answers to these specific comments below. We feel these changes greatly improved our study.*

Polar regions: Polar amplification is one of the main ways in which we expect the rate of warming to vary spatially across the globe, and you mention some of its drivers in the introduction. I think it would be important to include polar regions in this analysis. If the global temperature datasets use different definitions of the "surface" for sea ice areas (do they, or is this only true for SST datasets?), you could exclude polar regions from the

aggregated dataset but show global results for the individual datasets which include global surface temperatures.

Response: *We thank the reviewer for this comment. We have now expanded the region of analysis to include high latitudes and updated all Figures in the main and supplementary material, and different sections accordingly. As a result, there are now regions exhibiting multiple changes at high latitudes. That said, one must be cautious in interpreting these results given that surface temperatures are handled differently in sea ice areas. We added a word of caution to this effect in the discussion: The data in areas of sea ice are treated differently across datasets, which may affect our results at high latitudes [Menemenlis et al., 2025]. The five datasets used here all blend land surface air temperature with sea surface temperature, but differ in their treatment of sea ice regions. For example, NASA and HadCRUT use surface air temperature anomalies over ice-covered areas, while Berkeley uses SST measurements directly rather than as air temperature proxies [Hansen et al., 2010, Rohde and Hausfather, 2020, Morice et al., 2021].*

Land vs. Ocean: What percentage of land vs. ocean grid cells show a detectable acceleration in warming? This would be a meaningful and interesting piece of analysis to include: how much do we see "land amplification" reflected in these results? It would be helpful to see Figure 3 split into land/ocean parts.

Response: *We thank the reviewer for this comment and have updated Figure 3 to separate land and ocean. While land amplification is well established in terms of the overall magnitude of warming, changes in trend signs appear greater over the ocean, where both accelerations and decelerations occur, whereas land warming changes are more exclusively accelerating. Warming slowdowns were detected mainly over the ocean in the Southern hemisphere. We have added this nuance in our results and discussion.*

Data uncertainty: Some more discussion of the uncertainties in the underlying datasets would be helpful. What are the differences in regional trends between the different global temperature datasets? Do the datasets themselves report uncertainty ranges for grid-point temperature estimates? It may be worth noting that Menemenlis et al. (2025) find that there are large differences in spatial-average trends across SST datasets, but a much smaller difference in average trends across the global (combined land+ocean) datasets that you use here, because these global datasets use relatively similar SSTs.

Response: *A caveat to our analysis is that the set of land-ocean combined surface temperature datasets we examine here do not represent the full range and variation of regional and area-averaged SST trends seen across different SST datasets (e.g., Menemenlis et al. 2025). That is, the combined land-ocean datasets we examined tend to show much SST trend behavior that is more similar and consistent across the datasets than is seen across a wider sampling of SST datasets. We have expanded on this point in the discussion.*

Some datasets used here provide uncertainty ranges for grid-point estimates, but not all of them. A full analysis of grid-point uncertainties through our changepoint detection

framework would be a way to address this, and this is planned as a follow-up study. The degree of consistency across the five datasets examined provides a partial empirical assessment of robustness, with divergence across datasets in a given region serving as an indicator of larger uncertainty.

Internal variability: As you mention, processes in the Earth system give rise to long timescale memory, and known modes of variability such as the PDO have identifiable spatial patterns. The discussion section focuses on aerosols, and cites some examples of possible links between aerosol reductions and sections where change-points are detected. However, is there also a potential contribution to these patterns from modes of internal variability? While fully exploring this question is outside the scope of the paper, it warrants a more robust discussion.

Response: *We thank the reviewer for this comment and agree that modes of internal variability–including large-scale multidecadal variability–may be driving some of the changes we identified. We have added sentences in the discussion to highlight this possible driver.*

Quadratic trends: Figure S4 is a nice complement to the analysis in the main text. It corroborates many of the areas where piecewise warming accelerations are detected, and shows regional coherence. It's also interesting because it includes a significant decelerating trend in the cooling eastern Pacific, which is not detected in the change-point analysis. Why?

Response: *We thank the reviewer for this comment. We appreciate that the quadratic trend analysis is consistent with the changepoint analysis across most regions, and agree that it is somewhat less conservative in terms of detection, as it does not require estimating the timing of a changepoint and instead fits a smooth continuous acceleration or deceleration over the full record. The absence of a detected changepoint in the cooling eastern Pacific, despite the significant quadratic deceleration visible in Figure S4, reflects a fundamental difference between the two methods. The quadratic approach detects a gradual, continuous slowing of the cooling trend over the full period, which is well captured by a smooth polynomial fit. The change-point framework, by contrast, seeks an abrupt discrete shift between two distinct linear trends. If the deceleration in the eastern Pacific occurs gradually rather than abruptly, it may not produce a sufficiently sharp transition to be detected as a changepoint. The consistency between the two methods where both detect signals provides additional confidence in those results.*

Clarification: In general, when you write about "accelerations in warming," does this include any detectable increases in the rate of change of temperature, including from cooling to warming? My understanding is that some areas or grid cells, for example the North Pacific area as shown in Figure 4H, may have detectable negative trends during some part of the time period.

Response: *We thank the reviewer for this comment and agree with the reviewer that using "acceleration" is misleading when discussing a switch from cooling to warming. We have added a clarifying sentence in the results section to note that detected change-points represent statistically significant upward shifts in the local temperature trend, which in most regions*

corresponds to an acceleration of warming, but in some regions — particularly the North Pacific — may reflect a transition from cooling or near-zero trends to warming. We have audited the manuscript throughout and reserved "acceleration" strictly for cases where the pre-changepoint trend was already positive, and use "shift toward warming" for cases where the prior trend was negative or near-zero.

Line-by-line comments:

11-12: Slight clarification needed, as Rantanen & Laaksonen focused on the specific single event of the September 2023 warming "spike", and this sentence seems to refer to warming "spike" events in general.

Response: *We thank the reviewer for this comment. We have updated those lines in the introduction to be more accurate and make the distinction between the annual mean warming spike and the September 2023 spike.*

13-15: Here it would be worth noting the reduction in shipping tracks due to the 2020 International Maritime Organization regulation that limited sulfur in shipping fuel (e.g. Gettelman et al., 2024), as this would be specifically relevant to the 2023-2024 "spike".

Response: *Agreed. We have added a sentence in the manuscript to the effect of changes in shipping regulations.*

29-30: It's a common misconception that the primary reason for land/ocean warming contrast is due to variable heat capacities. While this is a factor, it is not the main factor, and the land-ocean contrast appears in the equilibrium response to warming (Manabe et al., 1991; Sutton et al., 2007).

Response: *We have rephrased this section to be more precise as: Land warms faster than ocean even at equilibrium, driven primarily by differences in evaporative cooling, atmospheric moisture transport, and lapse-rate feedbacks rather than heat capacity alone [Manabe et al., 1991, Sutton et al., 2007].*

31: As you mention high latitude processes here, it would be nice to include some analysis of high latitudes in this paper, even if it's only with individual datasets rather than an aggregate.

Response: *Agreed. We have updated our analysis to include high latitudes as mentioned in our response to your general comment. This addition led to the inclusion of an additional region in Figure 4, in east Greenland.*

42: Why did you choose to start in 1970?

Response: *We chose 1970 as our start date because it marks the well-documented global warming acceleration driven by a rapid change in global radiative forcing Bindoff et al. [2013].*

This period focuses on recent temperature trends while avoiding the structural breaks introduced by earlier data biases and sparsity. Starting here maximizes both data quality and detection power.

59: Instead of "experienced a change" do you mean "experienced a detectable change"?

Response: *Agreed, and modified.*

75-76: Is there a previous understanding / reference for why the Southern Ocean saw decelerations in the 1980s?

Response: *The temperature decelerations detected in the Southern Ocean in the 1980s are consistent with the well-documented surface cooling in that region during this period. Haumann et al. [2020] show that the Southern Ocean surface cooled between at least the early 1980s and early 2010s, attributing this primarily to increased wind-driven northward sea-ice transport that strengthened salinity-dominated surface stratification and reduced upward heat flux. A recent study also documents a cooling trend near the Drake Passage driven by freshwater forcing from melting Antarctic ice sheets [Kaufman et al., 2025], which may be related to the patterns we detect. We have added a discussion of these Southern Ocean results in the revised manuscript.*

80: Was there a particular threshold for the spatial extent, or for the level of consistency across datasets, of regions with detectable accelerated warming that you used to select these areas? And, can you clarify whether the timeseries shown in Figure 4 include the entire boxed area, or just the grid cells with detectable warming accelerations?

Response: *We selected areas where a change in the rate of warming is detected in multiple datasets. Yes, time series shown in Figure 4 correspond to the box average. We clarified in the figure caption.*

128: Interesting that Figure S4 includes significant negative quadratic trends in the eastern tropical Pacific, but no such decelerations are detected during the changepoint analysis. While other areas—including the major warming accelerating areas—show more similarities between the two methods. Why do you think this is? Would it be worth pointing this out?

Response: *See our response to the general comment about quadratic trends above.*

148-151: It doesn't seem necessary to completely exclude polar regions from this analysis: see comment above.

Response: *Agreed. See our response to general comment above.*

238: A major change in shipping regulations came in 2020, so would be relevant to warming trend since that year.

Response: *Agreed, we removed that part.*

247-249: Is it possible that some of the regional accelerations could have a significant con-

tribution from internal climate variability, not just external aerosol forcing? Though it's outside the scope of this study, one way to test this would be to perform an analysis similar to what you did in this manuscript on unforced climate model output.

Response: *We thank the reviewer for this comment, and agree this should be the focus of future work. We added a few sentences to this effect in the discussion.*

Figure 2: I understand "magnitude of regional changes in the rate of warming" to mean, for areas where piecewise changes are detected, the difference between the slopes of each piece.

Response: *That's correct. We specified this in Figure 2 caption for more clarity.*

Figure 4: It's interesting that Area H shows a change from cooling to warming trend—when I read "acceleration in warming" I assumed that these areas would all change from slow warming to faster warming, not cooling to warming. Would be worth clarifying.

Response: *Thank you for bringing this up. We have modified wordings throughout the paper to distinguish accelerations from shifts to warming.*

Figure S4 caption: Clarify which dataset used here? Also Berkeley?

Response: *Yes, also Berkeley. This is now clarified in the Supplementary Information.*

References: Gettelman, A., M. W. Christensen, M. S. Diamond, et al. "Has Reducing Ship Emissions Brought Forward Global Warming?" Geophysical Research Letters 51, no. 15 (2024): e2024GL109077. Manabe, S., R. J. Stouffer, M. J. Spelman, and K. Bryan. "Transient Responses of a Coupled Ocean–Atmosphere Model to Gradual Changes of Atmospheric CO2. Part I. Annual Mean Response." Journal of Climate. Journal of Climate 4, no. 8 (1991): 785–818. Menemenlis, Sofia, Gabriel Vecchi, Wenchang Yang, Stephan Fueglistaler, and Shiv Priyam Raghuraman. "Consequential Differences in Satellite-Era Sea Surface Temperature Trends Across Datasets." Preprint, In Review, February 4, 2025. Sutton, Rowan T., Buwen Dong, and Jonathan M. Gregory. "Land/Sea Warming Ratio in Response to Climate Change: IPCC AR4 Model Results and Comparison with Observations." Geophysical Research Letters 34, no. 2 (2007).

Reviewer 2

Main Comments:

This is a good and interesting manuscript, which would fit well into Nature Communications. The subject—rates of change of surface temperatures since 1970 across five gridded datasets—is of high scientific interest, particularly given the ongoing discussion about "global warming acceleration." The method (piecewise linear regression) is sound, and the presentation (text, figures, mathematical details) is generally adequate.

I recommend publication after three minor revisions are addressed. I first detail those three points, followed by several additional comments where a brief author response or small improvement would be beneficial.

Thank you for letting me review this work—I would be pleased to see the revised version.

Minor revisions:

(A) Uncertainty quantification for change-point parameters You perform piecewise linear regressions with an arbitrary number (m) of change-points, selecting m via the BIC criterion. This is essentially a statistical test for the number of linear segments, yielding slope estimates. The assumed data-generating process (piecewise linear plus AR(1) noise) is appropriate for temperature time series. However, the analysis would be strengthened by including uncertainty estimates (standard errors or confidence intervals) for the change-point times and slopes. I previously developed a related "break regression" method for m = 1 (Mudelsee 2009) using weighted least squares and moving-block bootstrap resampling of residuals. In my book (Mudelsee 2014, § 4.2.2), I examined bootstrap BCa confidence intervals and their coverage via Monte Carlo experiments. I recommend that you consider providing uncertainty ranges for your estimated change-point parameters—either by using the BREAKFIT tool (Mudelsee 2009, 2014) or by extending co-author Lanzante's software.

Response: *We thank the reviewer for this thoughtful suggestion and for pointing us to the BREAKFIT methodology. We respectfully clarify that our procedure is not a statistical test but a model selection approach, in which the number of changepoints and their locations are chosen jointly via BIC. In this framework, the selected model is treated as the best-fitting description of the data, analogous to how confidence intervals are not typically attached to a BIC-selected autoregressive model order. Attaching uncertainty ranges by conditioning on the selected number of changepoints m would miss the dominant source of uncertainty — the number of changepoints — and risks giving readers a false sense of precision. Furthermore, our scientific focus is on regional patterns rather than individual grid cell estimates, so uncertainty quantification at the grid cell level would not materially change our conclusions. We have nevertheless verified and tested the reliability of our approach through simulation experiments (Supplementary Material), which demonstrate low false detection rates and high power, and we have clarified the model selection framing in the revised manuscript.*

(B) Fairness toward prior work / citation completeness To acknowledge previous developments and broaden the readership's context, please cite the following two works:

Mudelsee M (2009) Break function regression: A tool for quantifying trend changes in climate time series. Eur Phys J Spec Top 174: 49–63.

Mudelsee M (2014) Climate Time Series Analysis: Classical Statistical and Bootstrap Methods, 2nd ed. Springer, Cham, xxxii + 454 pp.

Response: *We thank the reviewer for these helpful references, which we have added to the manuscript.*

(C) Spatial dependence This is admittedly a difficult issue. Nature exhibits memory not only in time (your AR(1) noise model) but also in space. Yet this aspect seems neglected (see line 157). Intuitively, ignoring spatial dependence may reduce the power of tests for post-1970 warming accelerations, but by how much? A Monte Carlo experiment could provide insight. At minimum, please discuss the implications of spatial dependence, and if feasible, include a quantitative analysis.

Response: *We thank the reviewer for raising this point. We note that this limitation is already acknowledged in the manuscript (line 241–243), where we recognize that treating grid cells independently ignores spatial correlations and that a space-time model could improve detection by borrowing strength from neighboring grid cells. We agree that a Monte Carlo experiment quantifying the power loss due to spatial dependence would be informative, but consider this beyond the scope of the current study and a valuable direction for future work.*

Additional comments:

Line 51: Provide a reference for long-memory models and clarify that their autocorrelation function decays hyperbolically, not exponentially as in short-memory (AR(1)) processes.

Response: *We clarified that for long-memory models the autocorrelation function decays as a power law and cite Muddelsee (2014) here.*

Line 101: Write "SNR = 2.6" (with spaces).

Response: *Done.*

Line 109: The statement "BIC penalty has been proven to perform best . . . " seems overly general. While you cite a reference, "best" depends on analytical goals (e.g., power, robustness to distributional shape or noise model). Please note such caveats.

Response: *We have specified in terms of Type I error and detection power.*

Lines 153–157: Clarify that you work with evenly spaced time series. (Uneven spacing is common in paleoclimate studies; see Mudelsee 2014 for discussion.)

Response: *We specify that we work with annual mean temperature series (i.e., evenly spaced data in time).*

Line 169: Allowing error-process parameters to change at the change-points is interesting but perhaps not parsimonious. Consider or at least discuss a stationary-error alternative.

Response: *We thank the reviewer for this comment. We agree that allowing error-process parameters to vary across segments adds model complexity, but we argue this flexibility is physically justified rather than arbitrary. Temperature variability and autocorrelation structure can change across climate regimes [Beaulieu and Killick, 2018, Beaulieu et al., 2024]. A stationary-error alternative would impose the same error structure across all segments, which*

could lead to misspecified residuals and unreliable BIC-based model selection. We have added a brief discussion of this tradeoff to the manuscript, noting that while a stationary-error model would be more parsimonious in some cases, our approach is designed to accommodate changing error structures that are commonly observed in climate records.

Line 179 ("Brute force"): For m = 1, the break method (Mudelsee 2009) remains computationally feasible even with bootstrap resampling. The change-point time can be restricted to observed time values, provided its uncertainty is similar to or larger than the temporal spacing near the break. The claim that PELT "guarantees the optimal solution" seems overstated unless the entire parameter hyperspace is explored—please clarify.

Response: *Yes, we do search the whole hyperparameter space in a computationally optimal way with the PELT algorithm. PELT guarantees the optimal solution in the sense that it finds the exact global optimum of the penalized likelihood over all possible change-point configurations, without approximation — this is what distinguishes it from heuristic search methods. However, this optimality is conditional on the assumed model (piecewise linear with AR(1) errors) and the chosen penalty function (BIC), not a claim about the broader parameter hyperspace. We have revised the manuscript to clarify this: "we use the pruned dynamic programming algorithm PELT [Killick et al., 2012], which guarantees the globally optimal solution for a given likelihood and penalty function, without resorting to heuristic search methods."*

Eq. (5), lines 191–192: Dimensional consistency is unclear. Parameters β_1 have units °C / decade, whereas σ_2 has units °C, making their ratio (the defined SNR) dimensional. Yet you present SNR as dimensionless (cf. line 101). Please revise or explain. In general, standard errors or CIs may convey estimation accuracy more transparently than SNR.

Response: *We thank the reviewer for catching this. We acknowledge that as written, the ratio $\frac{\beta}{\sigma}$ has units of year^{-1} (not decade), making the SNR dimensional. To correct this, we multiply each ratio by a reference time scale T (in years), yielding:*

$$SNR = |\frac{\beta_2 * T}{\sigma_2} - \frac{\beta_1 * T}{\sigma_1}|\sqrt{\frac{(\tau_1 - 1)(N - \tau_1)}{N}} \quad (1)$$

where T = 1 year, making both terms dimensionless. This is consistent with the interpretation of SNR throughout the manuscript and does not affect any of the numerical results, as T = 1 year was implicitly assumed in all computations. We have revised the manuscript accordingly.

References: Require careful reformatting (e.g., "ENSO," capitalization in no.6, incomplete author list in no.4 – who is "A.P."?).

Response: *Thank you for catching this. We have carefully checked the bibliography and corrected different issues with references.*

Figure 1: Distinguish "no data" (white) from "not detectable" (grey) instead of using one grey shade for both.

Response: *We thank the reviewer for highlighting this and have now missing data represented in grey and changes not detectable in white.*

Figure 3 caption: The phrase "x-axis" actually refers to the double horizontal axis showing percentages of detected change-points—please clarify.

Response: *Yes, the double horizontal axis shows percentages of detected changepoints. This is now specified in the caption.*

Figure 4: Panels B–H show m = 1. How many grid cells have m = 1, 2, or higher? A visual summary would be informative. Given your post-1970 focus, m = 1 likely dominates, in which case the break model (Mudelsee 2009) would be an excellent benchmark.

Response: *We thank the reviewer for this comment. We have added a new Table in the manuscript that summarizes the number of changes detected in the different datasets. With the expansion of the region of analysis to include high latitudes, there are indeed grid cells where* $m > 1$.

Supplementary Information line 56: "°C" should not be italicized (easily fixed in LaTeX).

Response: *Fixed.*

Supplementary Fig. S4: The quadratic-term results are interesting—perhaps emphasize them more in the main text.

Response: *We have added a few sentences about those results in the discussion section.*

Reviewer 3

This is a well-posed paper that uses piecewise fits to gridded surface temperature datasets to identify significant change points that can be interpreted as accelerations or deccelerations in the temperature time series at each grid point. The results demonstrate some regional accelerations, mostly in the midlatitudes of the Northern Hemisphere, and a mix of accelerations and deccelerations in the midlatitudes of the Southern Hemisphere. The topic is important and timely as there has been discussion of possible warming acceleration, and this paper provides a rigorous assessment of those regions in which this may be evident.

The analysis is sound and the authors have been clear in description of their methods and in highlighting limitations of the methods and checking, where possible, on the limitations. For example, the authors use multiple different gridded datasets to assess observational uncertainty. The authors are clear that their modelling of the surface temperature data does not account for long memory processes, and correctly note that this is more a limitation/caution over the oceans than over land areas. While they identify this as an issue for the North Pacific Ocean, this would also apply to the midlatitude acceleration/decceleration regions in the Southern Hemisphere, which are mostly over the Southern Ocean. The authors have

not really remarked on the Southern Hemisphere results here, which are in partial contrast to those in the Northern Hemisphere.

The paper is well written and provides sufficient detail to be reproduced. I can not identify any issues with the interpretation of the results, and their conclusions are all consistent with what they have shown. While that might seem unremarkable, it is refreshing to see. This paper surpasses the standards in the field, and is in my view, appropriate for publication, and makes a worthwhile contribution to current understanding of the expression of climate change.

Response: *We thank the reviewer for their positive assessment of our manuscript. We agree that the long memory caveat applies to the Southern Ocean midlatitude regions, just as it does to the North Pacific, and we have added a remark to this effect in the revised manuscript. Additionally, as suggested by another reviewer, we have expanded the analysis towards higher latitudes, which has allowed us to include more detailed discussion of the Southern Hemisphere results and their contrast with those in the Northern Hemisphere.*

References

C. Beaulieu and R. Killick. Distinguishing trends and shifts from memory in climate data. *Journal of Climate*, 31(23):9519–9543, 2018. doi: 10.1175/JCLI-D-17-0863.1. URL `https://journals.ametsoc.org/view/journals/clim/31/23/jcli-d-17-0863.1.xml`.

C. Beaulieu, C. Gallagher, R. Killick, R. Lund, and X. Shi. A recent surge in global warming is not detectable yet. *Communications Earth & Environment*, 5(1):576, 2024. doi: 10.1038/s43247-024-01711-1.

N. L. Bindoff, P. A. Stott, K. M. AchutaRao, M. R. Allen, N. Gillett, D. Gutzler, K. Hansingo, G. Hegerl, Y. Hu, S. Jain, I. I. Mokhov, J. Overland, J. Perlwitz, R. Sebbari, and X. Zhang. Detection and attribution of climate change: from global to regional. In T. F. Stocker, D. Qin, G.-K. Plattner, M. Tignor, S. K. Allen, J. Boschung, A. Nauels, Y. Xia, V. Bex, and P. M. Midgley, editors, *Climate Change 2013: The Physical Science Basis. Contribution of Working Group I to the Fifth Assessment Report of the Intergovernmental Panel on Climate Change*, pages 869–928. Cambridge University Press, Cambridge, United Kingdom and New York, NY, USA, 2013. doi: 10.1017/CBO9781107415324.022.

J. Hansen, R. Ruedy, M. Sato, and K. Lo. Global surface temperature change. *Reviews of Geophysics*, 48(4), 2010. doi: 10.1029/2010RG000345.

F. A. Haumann, N. Gruber, and M. Münnich. Sea-ice induced southern ocean surface buoyancy fluxes. *AGU Advances*, 1(1):e2019AV000132, 2020. doi: 10.1029/2019AV000132.

Z. Kaufman, E. Wilson, A. Purich, R. Beadling, and Y. Li. The impact of underestimated southern ocean freshening on simulated historical sea surface temperature trends. *Geophysical Research Letters*, 52(6):e2024GL112639, 2025. doi: https://doi.org/10.1029/2024GL112639. URL `https://agupubs.onlinelibrary.wiley.com/doi/abs/10.1029/2024GL112639`. e2024GL112639 2024GL112639.

R. Killick, P. Fearnhead, and I. A. Eckley. Optimal detection of changepoints with a linear computational cost. *Journal of the American Statistical Association*, 107(500):1590–1598, 2012.

S. Manabe, R. J. Stouffer, M. J. Spelman, and K. Bryan. Transient responses of a coupled ocean–atmosphere model to gradual changes of atmospheric CO2. Part I. annual mean response. *Journal of Climate*, 4(8):785–818, 1991. doi: 10.1175/1520-0442(1991)004⟨0785:TROACO⟩2.0.CO;2.

S. Menemenlis, G. A. Vecchi, W. Yang, S. Fueglistaler, and S. P. Raghuraman. Consequential differences in satellite-era sea surface temperature trends across datasets. *Nature Climate Change*, 15(8):897–903, 2025. doi: 10.1038/s41558-025-02362-6.

C. P. Morice, J. J. Kennedy, N. A. Rayner, J. P. Winn, E. Hogan, R. E. Killick, R. J. H. Dunn, T. J. Osborn, P. D. Jones, and I. R. Simpson. An updated assessment of near-surface temperature change from 1850: The HadCRUT5 data set. *Journal of Geophysical Research: Atmospheres*, 126(3):e2019JD032361, 2021. doi: 10.1029/2019JD032361.

R. A. Rohde and Z. Hausfather. The Berkeley Earth land/ocean temperature record. *Earth System Science Data*, 12(4):3469–3479, 2020. doi: 10.5194/essd-12-3469-2020.

R. T. Sutton, B. Dong, and J. M. Gregory. Land/sea warming ratio in response to climate change: IPCC AR4 model results and comparison with observations. *Geophysical Research Letters*, 34(2), 2007. doi: 10.1029/2006GL028164.

Response to Reviewers

"Space-time signatures of surface warming accelerations since 1970"
by Beaulieu, Johnson, Killick, Lanzante, and Knutson
Nature Communications

We thank the two reviewers for taking time to carefully re-assess our manuscript and for providing constructive comments. We have carefully revised the manuscript to address the reviewers' comments and prepared point-by-point responses. Below, we present the detailed responses to the reviewers' comments. Referees comments are in plain text and our responses in italic.

Reviewer 1

Main Comments:

The authors have addressed my concerns from the previous review. The additional clarifications and useful improvements to the discussion bring the manuscript into great shape for publication.

I have one remaining suggestion, which is to clarify the confusing wording on lines 16-20. There, the effect of ship track aerosol reductions is mentioned at the end of the 2nd paragraph of the introduction. Then, the 3rd paragraph begins, "another explanation for the exceptional temperatures..." and again invokes the effect of ship track aerosol reductions. The framing here is redundant and confusing.

Provided this small problem is corrected, I believe the manuscript is ready for publication.

Response: *We thank the reviewer for their positive assessment of our manuscript and for pointing out this remaining issue. We have removed the mention of ship track aerosols at the end of the third paragraph and only discuss ship tracks in the third paragraph.*

Reviewer 2

Main Comments:

My main concern remains unresolved.

The manuscript still does not provide uncertainty estimates (e.g. confidence intervals or standard errors) for the estimated change-point times and slope parameters. This is a central issue, as the main conclusions about regional warming accelerations depend directly

on the reliability of these estimates. Without such uncertainty quantification, the statistical strength of the results cannot be properly assessed.

I do not find the authors' argument convincing that uncertainty in the number of change-points would dominate and therefore make parameter uncertainty less relevant. At least for the frequent case of m = 1, uncertainty estimation for change-point timing and slopes would be straightforward and feasible with existing methods.

As an aside, I note that the distinction made between "model selection" and "statistical testing" does not remove the inferential nature of the procedure. The manuscript itself refers to detection rates and power, and the selected number of change-points is clearly subject to uncertainty given finite and noisy data.

For these reasons, I do not consider the manuscript sufficiently substantiated in its present form.

Response: *We thank the reviewer for this detailed critique and agree that uncertainty quantification is central to the reliability of our conclusions. We have revised the manuscript to address this concern more thoroughly, and we hope the additions below demonstrate that the results are indeed statistically well-substantiated.*

We have taken two complementary approaches to uncertainty quantification:

First, to address uncertainty in the underlying data, we downloaded and analyzed all members of the DCENT and HadCRUT observational ensemble (these are not available for the other datasets), allowing us to propagate data uncertainty through the changepoint detection procedure. This goes beyond what a single observational record would permit and provides a direct characterization of how sensitive the detected changepoints are to observational uncertainty. These results have been added to Supplementary Materials and are now mentioned in the results section.

Second, to address parameter uncertainty conditional on model structure of one changepoint, we computed confidence intervals for the changepoint timing for temperature averaged over key regions (Figure 4). These intervals are conditional on the presence of one changepoint (m=1). The regional results, which form the basis of the main conclusions about warming acceleration, are thus accompanied by appropriate uncertainty estimates.

We have chosen not to report grid-cell-level confidence intervals on the maps. Given the large number of locations and the spatial structure of the results, we believe the regional aggregates with associated CIs provide a clearer and more interpretable summary of parameter uncertainty than a map of per grid-cell intervals would. We have added a sentence in the Methods section making this choice explicit.

On the $m > 1$ case: we maintain that reporting parameter confidence intervals conditional on a fixed m, without acknowledging uncertainty in m itself, would give a misleadingly precise picture of overall estimation uncertainty. This is not a reason to avoid uncertainty quantification — as demonstrated above — but it is a reason to be explicit about what the reported intervals do and do not capture. We have revised the relevant passage in the manuscript to make this distinction carefully.

We hope these revisions — particularly the ensemble-based data uncertainty analysis and the regional confidence intervals for m = 1 — directly address the reviewer's central concern.

“Space-time signatures of surface warming accelerations since 1970”
by Beaulieu, Johnson, Killick, Lanzante, and Knutson
Nature Communications

This manuscript has now undergone two rounds of revision. Reviewers 1 and 3 were satisfied with the revisions after round 1. Reviewer 2 initially assessed the manuscript as requiring only minor revisions, but their concern about uncertainty quantification has persisted across rounds despite substantial additional analyses added in revision, including confidence intervals on regional averages and observational ensemble analyses. The manuscript also includes a Monte Carlo validation of the methodology that was part of the original analysis framework. In the current revision, we have further added a supplementary map of grid-cell changepoint timing uncertainty directly in response to Reviewer 2's latest assessment. In addition to regional confidence intervals for the changepoint timings, we have also added confidence intervals on the slopes before and after changes conditional on the changepoint timings. A key strength of our approach is its ability to detect multiple changepoints, making it particularly suited to a global and regional scale analysis where the number of changes is not known a priori. In their most recent assessment, Reviewer 2 acknowledged that the manuscript is “improved and scientifically interesting” and explicitly left the publication decision to the editor. Reviewer 4 was added, who qualified our analysis as ”interesting and sound” and addressing a question that is ”definitively of interest”. Reviewer 4 further commented that our ”assumptions are made transparent, are discussed and the choices seem well justified”. The responses below (in italic) addresses their remaining concern directly.

Reviewer 2 Comments:

Comment: The revised manuscript has improved, and I appreciate that the authors have now added confidence intervals for changepoint timings in the selected regional averages with one changepoint. This is a useful addition.

However, my central concern is only partially addressed. The manuscript still does not provide uncertainty estimates for the changepoint parameters at the grid-cell level, although Figures 1 and 2 are built around grid-cell changepoint timings and slope changes. In particular, no uncertainty estimates are provided for the slope parameters or for the slope changes, although these are central to the interpretation of warming acceleration.

Response: *We thank the reviewer for their careful and constructive assessment of the revised manuscript. In this revision, we have added a new supplementary map (Supplementary Figure S4) displaying confidence intervals for the timing of changes at the grid-cell level for all cells where a single changepoint was detected. These gridded uncertainty estimates are spatially consistent with the regional confidence sets presented in the main text and further reinforce our primary conclusions.*

Additionally, we have now included formal confidence intervals for the slope parameters of the regional averages in the main manuscript (Table 2). However, we respectfully decline to map slope parameter uncertainty to the individual grid-cell level. Because our framework detects multiple changepoints, fully mapping the uncertainty of the trends before and after each change at the grid scale would require generating a prohibitive number of additional global maps (at minimum, four separate maps representing the lower and upper bounds for both pre- and post-change slopes). Rather than clarifying the results, introducing such a large number of maps would compromise the readability of the manuscript and obscure the spatial patterns of warming acceleration.

We believe that presenting the spatial patterns of the central slope estimates in Figures 1 and 2—while cross-referencing them with the regional uncertainties (Table 2 and Figure 4) and newly added gridded timing uncertainties (Figure S4) strikes the balance between a rigorous treatment of uncertainty and clarity.

Comment: The newly added confidence intervals are conditional on a single-changepoint model and are provided only for regional averages. This is helpful, but it does not fully address the uncertainty of the main gridded results. Moreover, the method used for these confidence intervals relies on a submitted manuscript (co-authered by Rebecca Killick, a co-author of the present manuscript), and the present paper gives only a brief description of the procedure. I therefore find it difficult to assess how reliable these intervals are, including their coverage properties.

Response: *We appreciate this comment and recognize the importance of clarifying the statistical foundations of our uncertainty framework. The confidence sets employed here are rooted in well-established, peer-reviewed statistical methodologies conditional on a single-changepoint (Siegmund, 1988; Fang et al., 2020). These methods include Monte Carlo validations of coverage properties, as documented by Fang et al. (2020).*

To address the reviewer's concern regarding clarity, we have expanded the Methods section to explicitly outline the procedure and highlight these references, ensuring the description is entirely self-contained. We explicitly acknowledge in the text that these intervals are conditional on a single-changepoint model and are not designed to capture timing uncertainty in time series with multiple changepoints. Extending coverage to multiple changepoints remains an open research question in statistics and is the subject of ongoing development (e.g., Li and Killick, 2026).

Comment: The additional analyses based on observational ensembles are also useful, but they address data uncertainty rather than the parameter uncertainty of the changepoint model itself. This was not the main point of my previous concern.

Response: *We appreciate the reviewer clarifying this distinction. While we recognize that parameter uncertainty and data (observational) uncertainty are distinct, our decision to emphasize observational ensembles is rooted in the physical constraints of historical climate data. In spatial climate analysis—particularly in regions with sparse historical coverage—structural discrepancies between independent data products often represent the dominant source of un-*

certainty, potentially overriding parameter uncertainty.

By prioritizing observational ensemble analyses, we aimed to demonstrate that our identified warming accelerations are robust features of the Earth system rather than artifacts of a single gridded dataset's interpolation scheme. However, to ensure a balanced presentation, the revised manuscript now features both approaches: data uncertainty via the observational ensembles and parameter uncertainty via the newly added regional and gridded confidence intervals.

Comment: Overall, I regard the manuscript as improved and scientifically interesting. However, I still do not think that the uncertainty of the main changepoint results is quantified at the level needed for a fully convincing statistical assessment. I leave it to the editor to decide whether this remaining limitation is acceptable for publication in Nature Communications.

Response: *We would like to highlight the multiple converging lines of evidence that collectively support the robustness of our conclusions, including with respect to the slope changes. We assessed regional accelerated warming across multiple gridded datasets, accounted for observational uncertainty where possible, quantified changepoint parameter uncertainty for both regional averages and gridded changepoint timings, and validated the methodology via Monte Carlo experiments. Crucially, the spatial patterns of warming acceleration derived from the changepoint-based and quadratic approaches are in strong agreement, providing independent corroboration of the slope change results across grid cells. We are therefore confident that the conclusions drawn regarding warming acceleration are not sensitive to the absence of formal grid-cell-level slope uncertainty estimates. We also note that a key strength of our approach is its ability to detect multiple changepoints, making it particularly suited to a global and regional scale analysis where the number of changes is not known a priori and a single-changepoint assumption would be inappropriate. We believe that our thorough analysis and set of results fits well within the scope of Nature Communications.*

Reviewer 4 Comments:

Comment: The revised manuscript presents an interesting and sound analysis. The question whether global warming is accelerating is definitively of interest.

The authors make several assumptions, e.g., the piecewise linear model, AR[1] residuals, BIC penalty. All these assumptions are made transparent, are discussed and the choices seem well justified.

Response: *We thank the reviewer for their positive assessment of our manuscript and for recognizing that our core analytical assumptions are transparent, thorough, and well-justified.*

Comment: Choice of the penalty: The authors admit that the results are highly dependent on the choice of their penalty (BIC), which is definitively a weak point, which has been made transparent. However, I cannot follow their sensitivity study. I do not see the point in changing the penalty from (4m+4) to (3m+4). My first idea in this respect would be to

use the general penalized likelihood setup with a regularization parameter λ and use a few different λs in a sensitivity study. Maybe the authors can motivate their choice here.

Response: *We appreciate this opportunity to clarify the motivation behind our penalty structure. In the changepoint literature, the penalty is typically defined on a "per segment" basis, dictated by the number of additional parameters estimated when a new segment is added. A long-standing technical debate centers on whether the changepoint location parameters should be explicitly counted in this penalty term.*

A penalty coefficient of $4m$ *explicitly accounts for the location of the changepoints alongside the segment's structural parameters (the intercept, slope, and autocorrelation coefficient), whereas a coefficient of* $3m$ *excludes the location. By comparing the* $(4m + 4)$ *and* $(3m + 4)$ *formulations, we are testing the sensitivity of our results specifically against this fundamental structural distinction in changepoint parameter counting, rather than varying an arbitrary continuous regularization parameter* λ*. We have added a brief sentence to the Supplementary Information section 3.2 to explicitly motivate this choice.*

Comment: The quadratic model: The authors commented on their alternative model using a linear plus quadratic model that it does not provide a specific timing. While this is true, an accelerating trend does not necessary need a specific timing unless the model is bound to two distinct linear trends. The quadratic model shows acceleration w/o specific timing. I'd suggest to change the wording

Response: *We agree with the reviewer that an accelerating warming trend is physically meaningful without requiring an abrupt, specific onset timing. We have modified the wording in both the Main text and the Supplementary Information to remove any implication that the lack of a discrete changepoint timing is a limitation of the quadratic model, framing it instead as a complementary representation of an acceleration.*

Comment: Autocorrelated errors: It might be worth mentioning that the likelihood function does not easily factorize for autocorrelated errors. I suggest explaining this at least in the supplementary information.

Response: *We thank the reviewer for this comment. Because the residual errors in our model follow an autoregressive* $AR(1)$ *process, the resulting covariance matrix is non-diagonal. This prevents the joint likelihood function from factorizing into a simple product of independent marginal distributions. To clarify this for the reader, we have added a brief note in the Methods section explaining this non-diagonal structure and noting that the likelihood must be evaluated segment-by-segment to properly account for the conditional dependencies in the errors.*

Comment: Signal to noise ratio: I would have chosen another name for the concept shown as it gives a weighted difference of two SNRs.

Response: *We appreciate this feedback. We agree that using SNR for this metric can cause confusion with standard single-segment metrics. To more accurately reflect the nature of this*

metric, which measures the magnitude of a shift between two adjacent segments, we went back to the original terminology of "Changepoint Energy" (E) from the statistical literature throughout the revised manuscript and figures.

Comment: Regional averages: It is well explained how the average timing has been obtained when aggregating different data sets. But how is this done for the regions given in Fig. 4? Maybe I missed that. Is it the same grid-point wise analysis and then averaging of the timing? How are then CIs treated? Or are the time series averaged over the regions first? (Than the CI problem is clearer).

Response: *To clarify, the temperature time series across all grid cells falling within a designated geographic box are averaged *first* to construct a single, spatially aggregated regional time series. The changepoint analysis, parameter estimation, and formal confidence interval calculations are subsequently performed directly on the averaged time series. We have updated the caption of Figure 4 to make this completely explicit.*

Comment: Confidence intervals (CI): Confidence intervals are given conditioned on the choice of 1 break point. It might be noteworthy to explain why this is the case (penalized likelihood). Furthermore, the calculation of CIs has been deferred to an unpublished paper (60). I would expect that at least the idea should be formulated here. Furthermore, only the CIs of the timing are given (and only exemlarily for some interesting regional averages), not the CIs for the slopes as it has been asked by another reviewer.

Response: *We thank the reviewer for highlighting these points. We have addressed each aspect of this concern in the updated manuscript. The confidence sets employed here are rooted in well-established, peer-reviewed statistical methodologies conditional on a single-changepoint framework (Siegmund, 1988; Fang et al., 2020). These methods include rigorous Monte Carlo validations of empirical coverage properties, as documented by Fang et al. (2020). We explicitly acknowledge in the text that these intervals are conditional on a single-changepoint model and are not designed to capture timing uncertainty in cells with multiple changepoints. Extending to multiple changepoints remains an open research question in statistics and is the subject of ongoing development (e.g., Li and Killick, 2026).*

Confidence intervals for slopes and gridded timing: In this revised version, we have expanded our uncertainty framework to include formal confidence intervals for the slope parameters (both pre- and post-change) for the regional averages in the Main (Table 2). Furthermore, we have added a new global map in the Supplementary Information (Supplementary Figure S4) providing grid-cell level confidence intervals for changepoint timings across all cells where a single change was detected.

Comment: Smaller comments - how is the regridding done - l. 203 "... work have shown..." - the caption of Fig. 4 mentions a panel I which does not exist

Response: *Thank you for spotting these, they have been corrected. The regridding is done by averaging the small grids within larger 5x5 cells.*

References

Fang, X., Li, J., and Siegmund, D. (2020). Segmentation and estimation of change-point models: False positive control and confidence regions. The Annals of Statistics*, 48(3):1615 – 1647.*

Li, O. and Killick, R. (2026). Exact confidence set estimation in multiple changepoint problems. Submitted.

Siegmund, D. (1988). Confidence sets in change-point problems. International Statistical Review*, 56(1):31–48.*
